# Impact of overwork on self-assessed health of rural-to-urban migrants: Limitations of work incentives moderation effect and industry heterogeneity

**DOI:** 10.1371/journal.pone.0317588

**Published:** 2025-02-14

**Authors:** Zhaoxin Huo, Ya Wang

**Affiliations:** 1 School of Labor Economics, Capital University of Economics and Business, Beijing, China; 2 School of Tourism Management, South China Normal University, Guangzhou, China; AIIMS Jodhpur: All India Institute of Medical Sciences - Jodhpur, INDIA

## Abstract

Overwork is widely recognized as harmful to workers’ physical and mental health, yet studies focusing on income-driven rural-to-urban migrants are lacking. This research aims to explore the effects of working hours on the health of rural-to-urban migrants in China, examining the moderating role of work incentives and industry heterogeneity. Using 2018 China Labor-force Dynamics Survey (CLDS) data, we analyzed 3,475 valid samples with a binary logit model, categorizing working hours into comfortable work, tolerable work, moderate overwork, and severe overwork. Interaction and subgroup regression models were employed to examine the moderating effects of work incentives across industries. The results indicate that comfortable work does not improve health, while moderate overwork is harmful, with severe overwork having a greater negative impact. This effect is stronger for rural-to-urban migrants in capital-intensive industries compared to labor-intensive industries. Work incentives only mitigate health damage from overwork in labor-intensive sectors, but this effect disappears under severe overwork across all industries. This study contributes by highlighting the unique health impacts of overwork on income-driven rural-to-urban migrants and revealing the limitations of work incentives and industry differences, offering new insights into the relationship between employment and health.

## Introduction

Recent years have witnessed significant fluctuations in the working hours of global workers due to the COVID-19 pandemic and economic changes. Some industries have extended working hours to meet remote work demands, while others have reduced hours due to economic pressures. Overall, the average working hours remain high. In 2022, the global average was approximately 43.9 hours per week, well above the International Labour Organization’s maximum of 35 hours [1]. In China, the issue of overwork is particularly pronounced. According to the National Bureau of Statistics of China, the average weekly working hours for employees in enterprises have increased yearly since 2015, reaching 49 hours in 2023. This trend continued into 2024, with an average of 48.6 hours in June. While workers have the fundamental right to a safe and healthy working environment, excessive hours negatively impact work quality and other life aspects, such as physical and mental health and life satisfaction [2,3], and can even pose risks to life safety [4,5]. Therefore, the relationship between working hours and health is an important topic in labor economics and public health research.

Previous research has produced substantial findings on the relationship between working hours and physical and mental health. Many scholars have explored the effects of insufficient work or long hours on workers. For instance, studies by Milner et al. (2017), Guerin et al. (2021), and Wang et al. (2024) show that insufficient working hours fail to meet workers’ social and economic needs, adversely affecting their health [6–8]. Conversely, Sato et al. (2019) and Barck-Holst et al. (2022) found that moderate short working hours can benefit health [9,10]. Research on overwork consistently indicates that long working hours lead to mental and physical exhaustion [11,12], causing psychological issues like anxiety and stress [13], hindering recovery from fatigue [14,15], and affecting social activities, thus harming health. Some studies have also examined the potential moderating effects of work income and employment benefits on workers’ health [16,17], but findings are mixed. Xu et al. (2021) revealed that income can partially offset health losses from long working hours due to economic and mental incentives [18], while Zhang et al. (2023) argued that income is insufficient to fully counteract the health damage experienced by migrant workers [19]. This seemingly contradictory result may be attributed to differences within worker groups, which significantly influence the effectiveness of incentives. Therefore, when assessing the moderating role of incentives on the relationship between working hours and health, it is essential to consider the specific circumstances of different worker groups to develop more precise and effective health protection measures.

Current research on working hours and health primarily focuses on young laborers [20,21], healthcare workers [22], knowledge-intensive employees [23], and migrant workers [19], with less attention given to rural-to-urban migrants. However, this group is a unique product of the urban-rural dual structure and differs significantly from ordinary workers and mobile populations. Due to institutional factors and educational limitations, most rural-to-urban migrants work in the secondary labor market, facing long hours and high intensity, often leading to overwork. Their primary motivation for moving to cities is to pursue higher income and integrate into urban life, which may drive them to willingly extend their working hours at the expense of their health [1,24–26]. Given that rural-to-urban migrants constitute 30% of China’s labor market and are a key part of the workforce, it is urgent to explore the impact of work incentives on their health in relation to excessive working hours.

Theoretically, work incentives can improve income, social status, job security, and promotion opportunities [27,28], helping to balance the costs and benefits of excessive labor [29,30]. This suggests that rural-to-urban migrants with work incentives may experience better physical and mental health compared to those without. However, there are significant differences in physical, mental, and emotional demands among rural-to-urban migrants in different industries, leading to varying effects of work incentives on health [18,31]. Specifically, workers in labor-intensive industries primarily engage in physical labor, which is often repetitive and can negatively impact physical health [32]. In contrast, capital-intensive industries require higher cognitive and skill demands, leading to greater cognitive load and psychological stress per unit of time [33,34]. Therefore, even with corresponding work incentive mechanisms, the moderating effects on health from working hours can differ significantly across industries.

In summary, while previous studies have explored the moderating role of work incentives like income on the relationship between overwork and worker health, few have focused on income-driven rural-to-urban migrants. This study not only fills the academic gap in research on work hours and health among this population, but also uncovers the specific labor market needs of different migrant groups by examining the moderating effect of work incentives and industry heterogeneity. This study aims to systematically analyze the current working hours of rural-to-urban migrants and their impact on health, based on labor protection theory, to better understand the health risks of this unique group in the context of overwork. Moreover, we will explore work incentives as a moderating variable, investigating their compensatory effects on the relationship between working hours and health, with a heterogeneous analysis based on industry characteristics. Finally, this research will address key questions: (1) What impact do different working hour stages have on the physical and mental health of rural-to-urban migrants? Is overwork always detrimental? (2) Are there differences in the impact of working hours on health among rural-to-urban migrants across various industries? (3) In the context of overwork, can work incentives serve as a compensatory variable for the health of rural-to-urban migrants? What is the extent of this compensation, and are there health losses that cannot be reversed by work incentives? Addressing these issues will help governments and policymakers develop more effective labor time regulations and targeted health protection measures, thereby promoting the healthy development of the labor market and advancing sustainable socioeconomic progress.

## Research design

### Data

**Fig 1:**
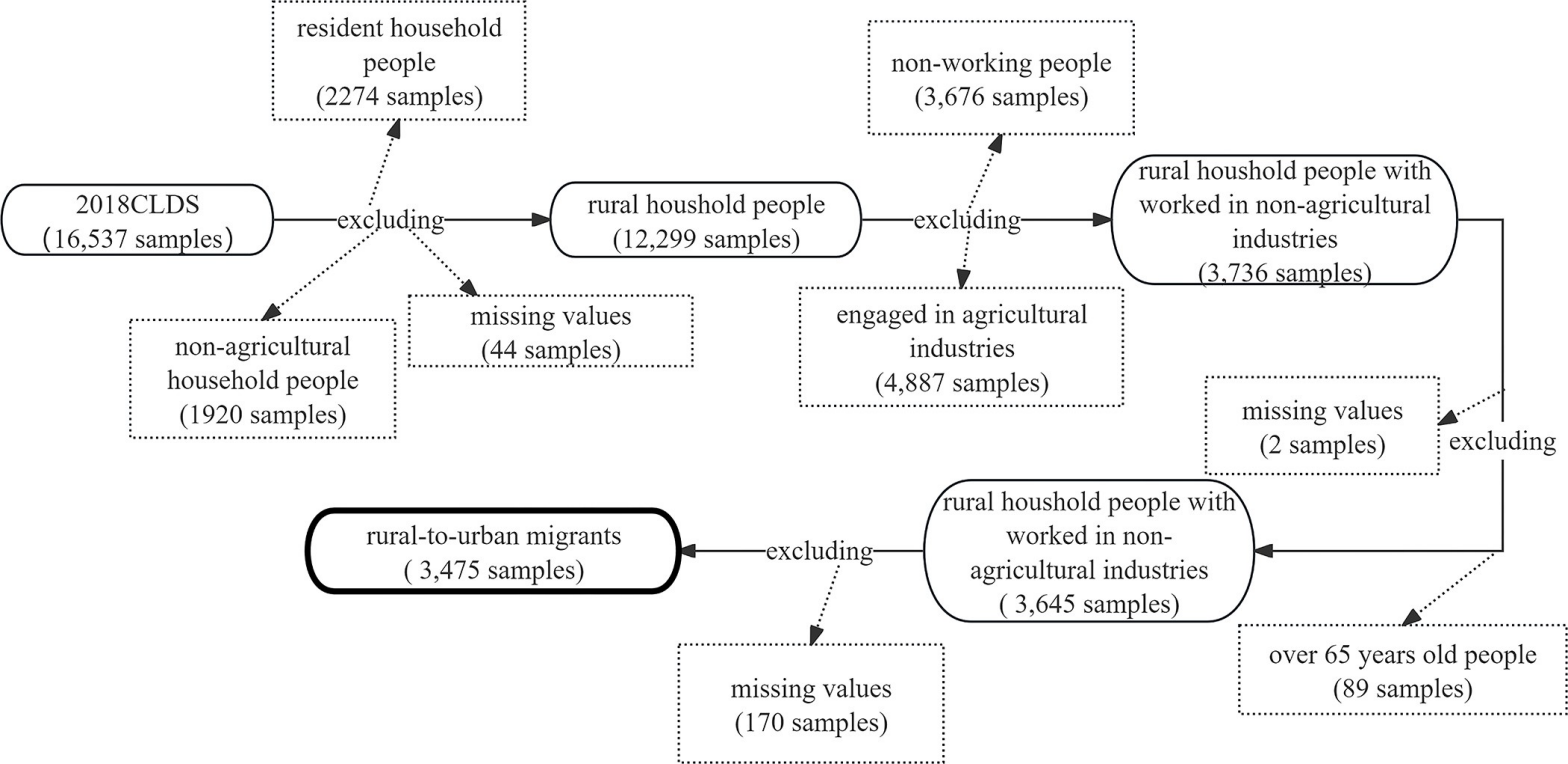
Sample screening flowchart.

Research on rural-to-urban migrants mainly draws from the China Migrants Dynamic Survey and the China Labor Force Dynamics Survey. For this study, which focuses on employment, data is sourced from the individual section of the 2018 China Labor Force Dynamics Survey (CLDS). Among the initial 16,537 samples, we identified rural residents based on the definition of rural-to-urban migrants [35] and confirmed they were employed in non-agricultural sectors, aged between 16 and 65. During this process, we excluded 4,238 samples with non-agricultural household registration and 8,563 individuals who had not worked or were engaged in agriculture, forestry, animal husbandry, or fishing in 2017, retaining only those aged 15 to 65. This resulted in a final sample of 3,645 rural-to-urban migrants. To ensure data completeness and accuracy, we further removed 170 samples with missing values across the variables, resulting in 3,475 valid samples (as shown in Fig 1). The 2018 CLDS individual survey questionnaire consists of nine sections: (1) Basic Information; (2) Educational Background; (3) Employment Status; (4) Work History; (5) Entrepreneurship; (6) Social Participation, Support, and Integration; (7) Labor Status; (8) Reproductive Health; and (9) Health Status. The selected variables for this study primarily focus on sections one, three, seven, and nine.

### Variables

After sample selection, key variables related to rural-to-urban migrants will be extracted, including physical and mental health, working hours, work incentives, and individual characteristics, as detailed below:

#### 1.Physical and mental health (dependent variable)

Physical and mental health is a complex concept encompassing various aspects, including physical well-being (such as illness and pain) and mental well-being (such as anxiety, depression, and stress). In this study, data on physical and mental health is derived from the Labor Status of the individual survey questionnaire, specifically part seven. The survey question regarding health (Question I7.5.1) asks, "How many times in the past year have you felt physically and mentally exhausted from work?" Frequent feelings of fatigue at work are often early feature of issues related to both physical and mental health [36]. Specifically, fatigue reflects decreased immune function, lack of sleep, or chronic stress, and prolonged physical and mental exhaustion may weaken the immune system and increase the risk of chronic diseases [37]. Thus, the frequency of feeling exhausted over the year can indirectly indicate workers’ health status. Respondents could choose from five options: “Every day,” “Several times a week,” “Several times a month,” “Several times a year or less,” and “Never.” Responses of “Every day,” “Several times a week,” and “Several times a month” indicate greater exhaustion and are defined as unhealthy (coded as 0, the reference group). Conversely, responses of “Several times a year or less” and “Never” indicate that rural-to-urban migrants do not feel exhausted, thus defined as healthy (coded as 1, the control group).

#### 2.Working hours (independent variable)

Data on working hours comes from the third section (Employment Status) of the questionnaire, including job identity and hours worked. For example, Question I3a.1 asks, “How many hours do you generally work in a week?” Participants respond with “__ hours.” Broadly, working hours refer to the total hours workers devote to work activities within a day or a week, including preparation before work, tasks after work, and paid work time [38,39]. According to the Labor Law of the People’s Republic of China, the standard workweek is set at 44 hours, allowing for an additional 1–3 hours of work daily, provided it does not exceed 36 hours monthly. Drawing on the work hour systems discussed by the International Labour Organization and various scholars [1,4], this study defines a reasonable working hour range for rural-to-urban migrants as 36–55 hours per week, categorized as tolerable work (the Reference Group). Working up to 35 hours per week is classified as comfortable work (Control Group 1); working 56–80 hours per week is considered moderate overwork (Control Group 2); and working more than 80 hours per week is classified as severe overwork (Control Group 4). Moderate and severe overwork are collectively referred to as overwork.

#### 3.Work incentives (moderating variable)

Work incentives address both the material and psychological needs of workers [40,41] and can be evaluated across dimensions like income, work environment, career prospects, and organizational recognition. Job satisfaction is used as a measure of work incentives because it reflects workers’ responses to these motivating factors [27,42]. When employees are satisfied with factors such as pay, promotion opportunities, and work conditions, it shows the effectiveness of these incentives. In this study, job satisfaction represents work incentives. Data for this variable is taken from the questionnaire, specifically question I7.3.11 on “overall job satisfaction.” Responses range from “very satisfied,” “somewhat satisfied,” “neutral,” “somewhat dissatisfied,” to “very dissatisfied.” In line with the classification of physical and mental health, responses of “very satisfied,” “somewhat satisfied,” and “neutral” are coded as 1, indicating the presence of work incentives, while “somewhat dissatisfied” and “very dissatisfied” are coded as 0, indicating their absence.

#### 4.Control variables

In addition, individual characteristics of rural-to-urban migrants also significantly impact their physical and mental health. Therefore, variables such as gender, age, educational attainment, marital status, and health status over the past year are included. Gender, age, educational attainment, and marital status are drawn from the first section (Basic Information) of the questionnaire, while health status over the past year is from the ninth section (Health Status), based on the question “Have you been hospitalized in the past year as diagnosed by a doctor?” A “yes” response indicates poor health, while a “no” response indicates relatively good health over the past year.

## Methods

After data preparation, statistical analysis should be conducted. All analyses, including descriptive statistics and regression analyses (binary logit regression and interaction analysis), will be performed in Stata 15.1. (1) Descriptive Statistics: This includes calculating the mean, standard deviation, skewness, and kurtosis of each variable. The focus is on examining the working hours and physical and mental health of rural-to-urban migrants. Working hours are categorized into four groups: comfortable work, tolerable work, moderate overwork, and severe overwork. The health of workers in each group are analyzed. Additionally, based on differences in the proportions of production factors like resources, labor, technology, and capital, workers are classified into labor-intensive and capital-intensive industries, and their health is observed across different work hours in each industry. (2) Regression Analysis: Since health is a binary variable, a binary logit model is used as the main regression model. Before regression, variance inflation factor (VIF) tests are conducted to eliminate multicollinearity, and the likelihood ratio test and Hosmer-Lemeshow goodness-of-fit test are used to verify the validity and fit of the regression results [43]. Fisher’s test is applied to assess the significance of differences across groups during subgroup regressions [44,45]. The odds ratios (ORs) and standard deviations for comfortable work, moderate overwork, severe overwork, and work incentives are reported. Statistical significance for all analyses is set at 0.05.

### Addressing potential bias

Although regression analysis can verify relationships between variables, some biases are inevitable. (1) Sample selection bias: During sample selection, we inevitably excluded rural-to-urban migrants who were unemployed or for whom physical and mental health data could not be collected, which may introduce inherent sample bias. However, in the original survey, a multi-stage, stratified, probability sampling method proportional to labor force size was employed to ensure randomness and diversity in the sample, minimizing the potential effects of selection bias. (2) Omitted variable bias. In empirical analysis, the physical and mental health of rural-to-urban migrants may be linked to their characteristics, such as age and education, which are difficult to fully control. To address potential omitted variable bias, we include variables like age, gender, educational attainment, marital status, and previous year’s health in the model to minimize bias from unobserved factors.

### Ethical statement

This study used publicly available micro-survey data national wide and did not require the ethical review board approval, as it did not involve direct data collection from human or animal subjects. The data obtained from publicly available sources were anonymized and collected by ethical standards. No identifiable personal information was accessed. There are no conflicts of interest to declare.

## Results

### Description of the variables

Table 1 reports the measurement criteria and descriptive statistics for all variables in this study. The sample of rural-to-urban migrants has a slightly higher proportion of men (mean gender value = 0.57), with an average age of 42 years. Most are married (over 80%), and the majority have a middle or high school education. More than half are satisfied with their current job, indicating that their work is motivating. Over the past year, most reported good health (lagged health mean = 0.94). Key variables show that rural-to-urban migrants have relatively low physical and mental health levels (mean = 0.43), with an uneven distribution (right-skewed). Overwork is also common, with a mean value exceeding 2. This data highlights the multiple challenges faced by China’s lower-tier labor force, especially rural-to-urban migrants, in terms of living conditions, work pressure, and health.

**Table 1:** Variable definitions and sample descriptive statistics.

Variables	Definition	Mean	Std.	Minimum	Maximum	Skewness	Kurtosis
Physical and Mental Health	Healthy = 1, Unhealthy = 0	0.43	0.50	0	1	0.27	1.07
Working Hours	Comfortable work = 1, Tolerable work = 2, Moderate overwork = 3, Severe overwork = 4	2.34	0.91	1	4	-0.01	2.11
Work Incentives	Satisfied with job = 1, dissatisfied = 0	0.57	0.50	0	1	-0.28	1.08
Gender	Male = 1, Female = 0	0.57	0.50	0	1	-0.27	1.07
Age	continuous variable	41.64	11.80	15	65	-0.07	2.07
Marital status	In marriage = 1, out of marriage = 0	0.81	0.33	0	1	-2.26	6.10
Educational Levels	Junior high school and below = 1; high school/secondary = 2; college and above = 3	1.44	0.71	1	3	1.30	3.21
Lagged- Health	Healthy = 1, Unhealthy = 0	0.94	0.23	0	1	-3.88	16.06

**Fig 2:**
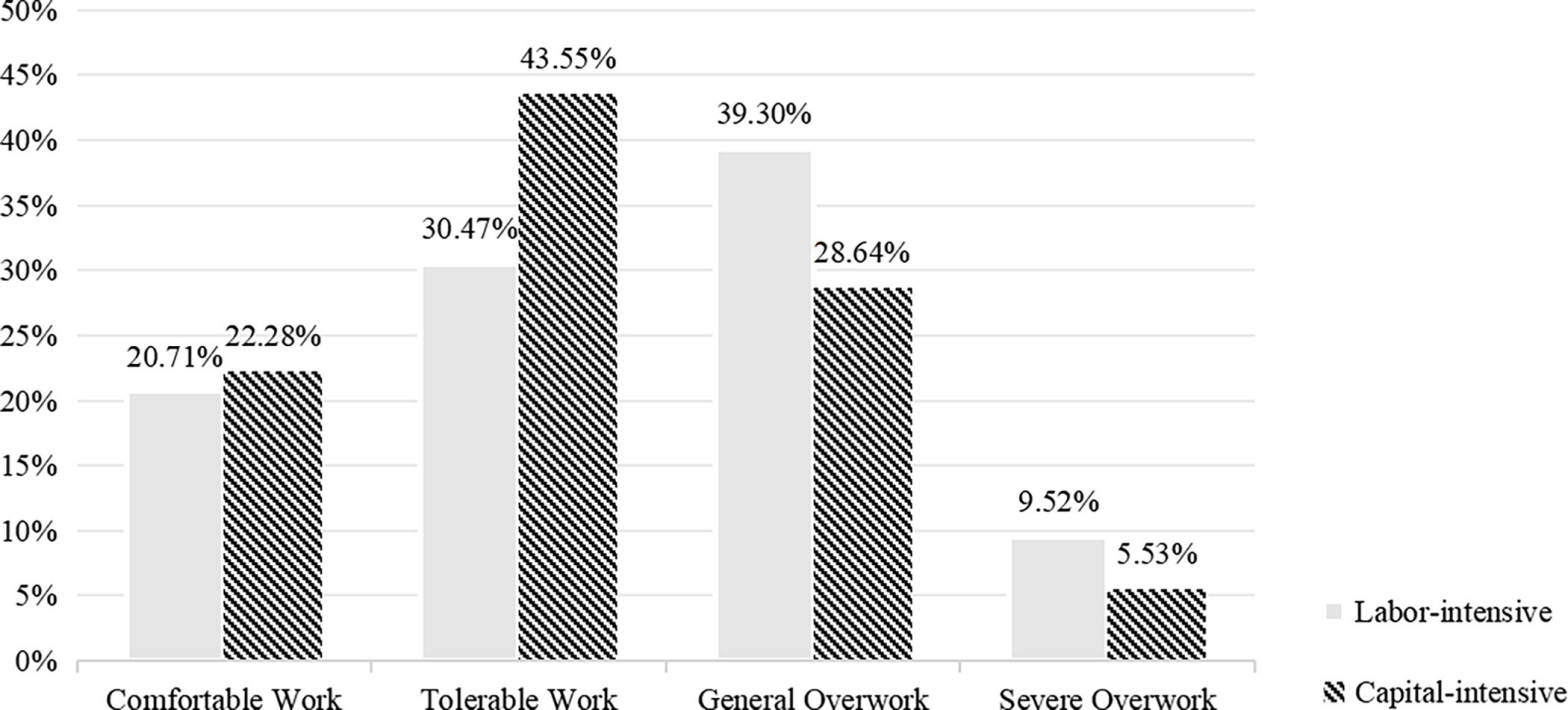
Proportion of working hours for rural-to-urban migrants in different industries.

Fig 2 shows the distribution of working hours for rural-to-urban migrants in labor-intensive and capital-intensive industries. The proportion of rural-to-urban migrants engaged in comfortable work is similar in both sectors, around 20%. In capital-intensive industries, 43.55% of rural-to-urban migrants are involved in tolerable work, significantly higher than the 30.47% in labor-intensive industries. The share of rural-to-urban migrants experiencing moderate overwork in labor-intensive industries rises to 39.3%, while it decreases to 28.64% in capital-intensive industries. The proportion of severe overwork is the lowest in both sectors, yet it remains higher among labor-intensive rural-to-urban migrants. This indicates that rural-to-urban migrants in labor-intensive industries primarily engage in overwork, whereas those in capital-intensive industries are more likely to experience comfortable and tolerable work conditions.

**Fig 3:**
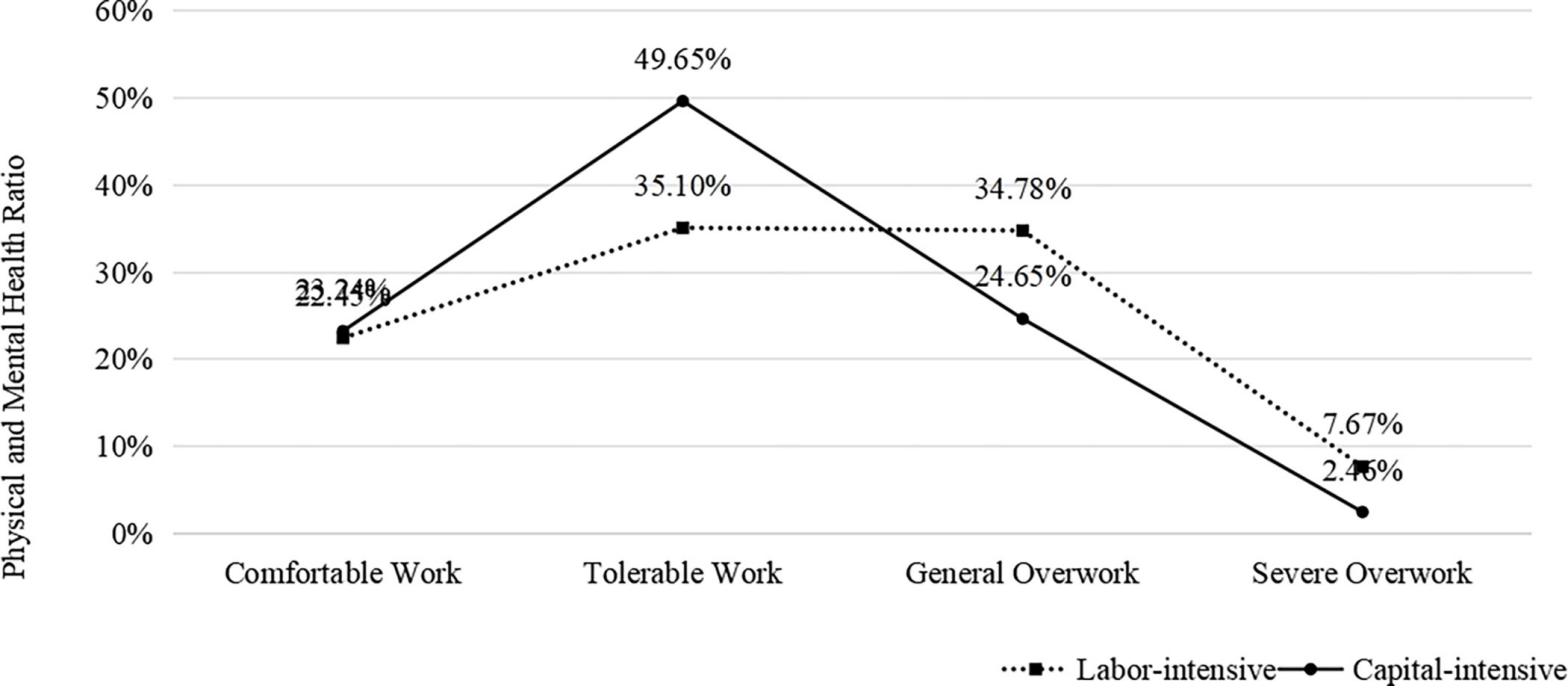
Health proportion of rural-to-urban migrants in different industries across working hours.

Further analysis reveals that the physical and mental health status of rural-to-urban migrants varies across industries with changes in working hours (as shown in Fig 3). In the comfortable work stage, the health proportion for rural-to-urban migrants in both labor-intensive and capital-intensive industries is around 22%-23%. In the tolerable work stage, the health proportion in capital-intensive industries approaches 50%, while it drops to 35.1% in labor-intensive industries. In the moderate overwork stage, health in capital-intensive industries declines significantly to 24.65%, while labor-intensive industries remain relatively higher at 34.78%. In the severe overwork phase, both sectors see a substantial decline, but the health proportion in capital-intensive industries (2.46%) is much lower than in labor-intensive industries (7.67%). Overall, the “working hours—health” curves for both industries exhibit a non-linear pattern. In the comfortable and tolerable work stages, the health proportion of rural-to-urban migrants is higher in capital-intensive industries. However, as working hours extend into the overwork stage, health in capital-intensive industries drops sharply, significantly below that in labor-intensive industries. This indicates that capital-intensive industries provide better health protection under moderate working conditions. In contrast, labor-intensive industries may have a higher tolerance for long working hours, resulting in relatively less health deterioration under high-intensity labor.

Next, we will use econometric software to empirically analyze the relationship between working hours and the physical and mental health of rural-to-urban migrants, as well as explore industry heterogeneity.

### Impact of working hours on physical and mental health

Table 2, Model 1 presents the estimated regression results for the impact of working hours on the physical and mental health of rural-to-urban migrants. The results indicate that compared to tolerable work, overwork has a significant negative effect on their health. Specifically, the probability of health among rural-to-urban migrants engaged in moderate overwork decreases by 34.9% (1–0.651), while in the case of severe overwork, the decline is 46.8% (1–0.532). Additionally, factors such as age and years of education positively contribute to improving health, which aligns with common expectations. Model 2 presents the results of the industry heterogeneity analysis. Compared to tolerable work, moderate overwork reduces the probability of health for rural-to-urban migrants in labor-intensive industries by 33.1% (1–0.669) and by 40.9% (1–0.591) in capital-intensive industries. For severe overwork, the health probability in labor-intensive industries decreases by 40.7% (1–0.593), while in capital-intensive industries, it declines by a significant 77% (1–0.230). This indicates that overwork has a greater negative impact on health in capital-intensive industries than in labor-intensive ones, with the difference more pronounced under severe overwork. This may be due to the higher mental strain and work pressure faced by workers in capital-intensive industries, leading to more serious health damage.

**Table 2:** Impact of working hours on health of rural-to-urban migrants and industry heterogeneity.

	Physical and Mental Health (Model 1)			Physical and Mental Health (Model 2)					
	All			Labor-intensive			Capital-intensive		
	odds radio	Std. Error	p>|Z|	odds radio	Std. Error	p>|Z|	odds radio	Std. Error	p>|Z|
**Benchmark Group:Tolerable work**									
Comfortable work	0.909	0.088	0.324	0.931	0.102	0.516	0.854	0.184	0.464
Moderate overwork	0.651	0.055	0.000	0.669	0.063	0.000	0.591**	0.124	0.012
Severe overwork	0.532	0.073	0.000	0.593	0.087	0.000	0.230***	0.104	0.001
**Control Variables**									
Gender	0.989	0.070	0.878	0.939	0.073	0.415	1.340	0.266	0.140
Age	1.009	0.004	0.013	1.012	1.012	0.002	0.989	0.010	0.259
Educational Attainments	1.233	0.068	0.000	1.269	0.083	0.000	1.123	0.131	0.319
Marital status	0.975	1.233	0.836	0.896	0.127	0.437	1.323	0.351	0.291
Lagged-Health	1.146	1.146	0.373	1.164	1.164	0.373	1.099	0.389	0.789
**Cons**	1.009	0.111	0.001	0.509	0.109	0.001	0.446**	0.501	0.815
**Samples**	3475			2878			597		
**LR chi2(8)**	71.84			56.60			21.54		
**Hosmer-Lemeshow**	12.53			3.82					
**Pseudo R** ^**2**^	0.0151			0.0144			0.0261		
**Fisher Combined Tests**	-			Significant difference in coefficients					

### The moderating effect of work incentives and industry heterogeneity analysis

To facilitate the calculation of the moderating effect of work incentives under different level of overwork, this study categorizes the working hours of rural-to-urban migrants into two groups: those experiencing overwork (T≥56) and those experiencing severe overwork (T>80). The moderating effects of work incentives and industry heterogeneity analysis are conducted for each group.

#### 1. The moderating effect of work incentives under overwork and industry heterogeneity analysis

Table 3, Model 1 shows the binary Logit regression results for overwork (T≥56), work incentives, and their interaction (0 for no work incentives and 1 for overwork with work incentives) on the health of rural-to-urban migrants. Taking non-overwork as the baseline, overwork without work incentives leads to a 44.7%(1–0.553) decrease in health probability. However, with work incentives, this decline is reduced to 8.1% [(1−0.553)−(1.366−1)]. This indicates that work incentives somewhat mitigate the negative impact of overwork on the health of rural-to-urban migrants, but do not completely eliminate the health risks associated with overwork. Thus, even with incentives, rural-to-urban migrants still face a certain level of health risk.

**Table 3:** Moderating effect of work incentives under overwork and industry heterogeneity.

	Physical and Mental Health (Model 1)			Physical and Mental Health (Model 2)					
	All			Labor-intensive			Capital-intensive		
	odds radio	Std. Error	p>|Z|	odds radio	Std. Error	p>|Z|	odds radio	Std. Error	p>|Z|
Overwork (T≥ 56)	0.553	0.062	0.000	0.569	0.069	0.000	0.474	0.142	0.013
Work incentives	1.757	0.169	0.000	1.790	0.193	0.000	1.639	0.362	0.025
Overwork*work incentives	1.366	0.197	0.031	1.364	0.215	0.049	1.386	0.516	0.380
**Control Variables**	Control	-	-	Control	-	-	Control	-	-
**Cons**	0.332	0.084	0.000	0.294	0.083	0.000	0.595	0.346	0.3782
**Samples**	3475			2878			597		
**LR chi2(8)**	171.15			146.59			28.71		
**Hosmer-Lemeshow chi (8)**	5.10			4.75					
**Fisher test**	-			Significant					
**Pseudo R** ^**2**^	0.0360			0.0373			0.0347		

Model 2 analyzes the heterogeneous effects across different industries. In labor-intensive industries, without work incentives, overwork leads to a 43.1%(1–0.569) decrease in health probability, while with work incentives, this decline reduces to 6.7%[(1−0.569)−(1.364−1)]. However, in capital-intensive industries, overwork results in a 52.6%(1–0.474) decrease in health probability, but the moderating effect of work incentives is not significant, indicating that work incentives do not mitigate health issues for rural-to-urban migrants in capital-intensive industries. In summary, work incentives only mitigate the negative health effects of excessive labor among rural-to-urban migrants in labor-intensive industries. This may be because rural-to-urban migrants in labor-intensive industries have a stronger need and desire for income increases and job security compared to those in capital-intensive industries.

#### 2. The moderating effect of work incentives under severe overwork and industry heterogeneity analysis

Model 1 in Table 4 presents the binary Logit regression estimates for severe overwork (T>80), work incentives, and their interaction (0 indicates no work incentives, and 1 indicates severe overwork with work incentives) on health. The analysis shows that the interaction between severe overwork and work incentives is not significant, and the industry heterogeneity analysis yields similar results. This indicates that the physical exhaustion and mental fatigue caused by severe overwork exceed what incentives can compensate for. Therefore, when rural-to-urban migrants are in a state of severe overwork, work incentives have no moderating effect on their health, regardless of the industry.

**Table 4:** Moderating effect of work incentives under severe overwork and industry heterogeneity.

	Physical and Mental Health (Model 1)			Physical and Mental Health (Model 2)						
	All			Labor-intensive			Capital-intensive			
	odds radio	Std. Error	p>|Z|	odds radio	Std. Error	p>|Z|	odds radio		Std. Error	p>|Z|
Severe Overwork (T>80)	0.683	0.139	0.062	0.791	0.172	0.280	0.248		0.160	0.031
Work incentives	2.059	0.154	0.000	2.127	0.175	0.000	1.783		0.325	0.002
Overwork*work incentives	0.928	0.244	0.775	0.827	0.229	0.494	1.861		1.673	0.490
**Control Variables**	Control	-	-	Control	-	-	Control		-	-
**Cons**	0.252	0.062	0.000	0.222	0.061	0.000	0.501		0.287	0.227
**Samples**	3475			2878			597			
**LR chi2(8)**	145.92			126.22			27.12			
**Hosmer-Lemeshow chi (8)**	17.32			5.56						
**Fisher test**	-			Significant						
**Pseudo R** ^**2**^	0.0307			0.0322				0.0328		

This research reveals that rural-to-urban migrants are generally overworked, with overwork significantly harming their physical and mental health. Those in severe overwork, especially in capital-intensive industries, face greater health risks. While work incentives can mitigate the health impact of overwork in labor-intensive industries, they have no significant moderating effect under severe overwork in any industry.

## Discussion

Although many previous studies have explored the impact of working hours or overwork on workers’ physical and mental health, few have focused on rural-to-urban migrants driven primarily by economic gain. Even fewer have conducted empirical research on how material and mental incentives moderate the relationship between working hours and health for this group. Using data from the 2018 CLDS, this study deeply analyzes the current state of working hours, work incentives, and health among rural-to-urban migrants. It also examines the proportion of this group’s health across different working hours. Additionally, we divide the sample into capital-intensive and labor-intensive industries to explore industry-specific differences in the relationship between working hours and health. For the empirical analysis, a binary Logit model is employed as the baseline regression to examine changes in the health of rural-to-urban migrants under varying working hours. Interaction models are then introduced to investigate the moderating role of work incentives.

Firstly, this study finds that rural-to-urban migrants in capital-intensive industries are more likely to experience tolerable work, while those in labor-intensive industries are more prone to moderate overwork. Several scholars have also pointed out that individuals engaged in manual labor—primarily employed in construction, manual manufacturing, wholesale and retail, transportation, accommodation, and food services—are more vulnerable to overwork [46,47]. This phenomenon is often explained by the flexible working hours in these industries, which are closely tied to market fluctuations and hard to regulate under fixed working hour systems [48,49], rather than addressing the core factors like industry production methods and workforce characteristics. Specifically, capital-intensive industries rely on advanced technology and equipment, reducing the physical demands on labor [50], thus shortening working hours. In contrast, labor-intensive industries rely heavily on manual labor, with high labor demand and repetitive tasks [51], making rural-to-urban migrants in these industries more susceptible to long working hours. Analyzing the causes of overwork from the perspective of industry attributes provides a more accurate understanding of the problem and offers strategies for fundamentally addressing it.

Secondly, rural-to-urban migrants in labor-intensive industries have a higher tolerance for overwork, with less health damage compared to those in capital-intensive industries. Empirical results further confirm that moderate overwork significantly reduces rural-to-urban migrants’ health, with the impact of severe overwork being even more pronounced, especially in capital-intensive industries. This phenomenon has yet to receive much academic attention, and its causes remain underexplored. In terms of job nature, rural-to-urban migrants in capital-intensive industries often in high-skill, senior management positions that require significant mental labor [52]. Prolonged overwork sharply increases both physical and mental strain, posing serious challenges to their health [53]. In contrast, although rural-to-urban migrants in labor-intensive industries face physically demanding work, the tasks are often repetitive, leading to lower mental strain and psychological pressure, resulting in less health damage [54,55]. This deepens the academic understanding of the consequences and factors of overwork among rural-to-urban migrants, providing important reference information for the labor market.

Furthermore, while work incentives can partially offset the health risks of overwork in labor-intensive industries, their effects are limited and do not fully counteract the negative impacts. This finding is innovative. The compensatory effect of work incentives primarily depends on the trade-off between the additional benefits from overwork and the resulting physical and mental harm. In capital-intensive industries, higher income and better social security lead rural-to-urban migrants to prioritize mental pursuits such as leisure and socializing [56]. Even when incentives match the demands of overwork, they perceive their marginal benefits as insufficient, limiting the moderating effect on health damage. In contrast, rural-to-urban migrants in labor-intensive industries have weaker job security and tend to follow a wage-based principle [57]. Thus, they are more likely to accept work incentives as compensation when facing overwork. Empirical research shows that work incentives can reduce the negative impact of overwork on health from 43.1% to 6.7%. However, it is important to note that the moderating effect of work incentives does not completely eliminate the negative impact of overwork on health. Therefore, we need to explore more comprehensive and effective incentive measures and seek new ways to promote physical and mental well-being. This helps guide businesses and workers to reasonably adjust working hours and intensity, promoting the healthy and stable development of the labor market.

Finally, the compensatory effect of work incentives is not significant under severe overwork. While many scholars have recognized the serious consequences of excessive overtime, including mental illness [58], suicidal behavior [59], and cases of overwork-related death [60], there remains insufficient differentiation regarding the extent of overwork and its specific outcomes. This study expands on the investigation of overwork by further differentiating between moderate and severe overwork, with a particular emphasis on cases where weekly working hours exceed 80. It analyzes the impact of severe overwork on the health of rural-to-urban migrants across different industries, revealing the dangers of severe overwork and the limitations of work incentives. This is also one of the core findings of this research. Severe overwork is accompanied by heightened work and competitive pressure, leading to significant negative effects on health. Even with incentives, these pressures may paradoxically increase, causing irreversible harm to the health of rural-to-urban migrants. Therefore, severe overwork should be firmly eradicated.

## Conclusion

This research leads to the following conclusions: Rural-to-urban migrants commonly face overwork, particularly in labor-intensive industries, while those in capital-intensive industries mostly experience tolerable work. As working hours increase, their health ratio initially improves but then declines. Specifically, rural-to-urban migrants in capital-intensive industries show better health under comfortable or tolerable work, whereas those in labor-intensive industries exhibit greater tolerance for moderate overwork. Regression analysis further reveals that comfortable work does not improve health, while moderate overwork significantly harms it, with severe overwork posing greater risks, especially in capital-intensive industries. Work incentives can mitigate the health impact of overwork in labor-intensive industries, but they are ineffective in capital-intensive ones, highlighting the need for differentiated labor time regulations and health protection measures for rural-to-urban migrants. Under severe overwork, the moderating effect of work incentives is insignificant, and the damage to rural-to-urban migrants’ health becomes irreversible. Thus, preventing severe overwork should be the top priority in labor time management. In a broader socioeconomic context, these findings offer theoretical support for governments and businesses to formulate more reasonable labor management and health protection policies, promoting a healthier labor market and sustainable socioeconomic progress.

To address the issues identified, this paper proposes the following targeted recommendations: (1) Focus on work incentives for rural-to-urban migrants in labor-intensive industries under overwork. While overwork often leads to a decline in health for this group, appropriate work incentives can help offset these effects. The key is to ensure that incentives are well-designed to minimize the negative impact of overwork, and even transform it into a positive factor for health. The government should play an active role in encouraging and guiding companies to establish scientific and reasonable compensation systems, covering both material aspects like salary and benefits, as well as psychological support and respect, ensuring rural-to-urban migrants’ smooth integration and development in urban areas. (2) Leverage smart technologies to improve production efficiency and reduce moderate overwork in capital-intensive industries. Companies should actively adopt new technologies to reduce reliance on manual labor. When overwork is unavoidable, companies should provide higher compensation and benefits to acknowledge employees’ efforts. Additionally, they should offer psychological support and health services to help employees manage stress and fatigue. (3) Fully prevent severe overwork across all industries. At the government level, labor market regulation should be strengthened, with stricter guidelines on working hours and clear legal boundaries between reasonable and excessive labor. A robust supervision and penalty system should be established to protect workers’ rights. At the individual level, workers should enhance their skills through formal education and training, increasing their job security and reducing the risk of overwork due to skill gaps or information asymmetry.

Like other studies, this paper has some limitations. First, although it uses the latest data from the CLDS series of surveys, it does not incorporate data from the previous four waves, which could provide a more accurate understanding of changes in rural-to-urban migrants’ working hours and health. Future research could address this. Second, due to space constraints, the paper does not explore the potential complex effects of comfortable work on health, which could be expanded to include research on flexible employment and health. Finally, limited data availability prevented separate analysis of the effects of material and psychological incentives on the relationship between working hours and health among rural-to-urban migrants. Future studies could explore this in greater depth to gain a more comprehensive understanding of incentive mechanisms.

## Supporting information

S1 File (PDF)

S2 File (DO)

S3 File (DOCX)

S4 File (ZIP)

S5 File (DTA)

S6 File (XLSX)
