# Supplementary material for: Impact of overwork on self-assessed health of rural-to-urban migrants: Limitations of work incentives moderation effect and industry heterogeneity
Source: PLoS One. 2025 Feb 14;20(2):e0317588. doi: 10.1371/journal.pone.0317588 (PMC11828385; doi:10.1371/journal.pone.0317588)
Supplement: S1 File — (PDF) [file pone.0317588.s001.pdf]

中国劳动力动态调查（CLDS）

调查手册

中山大学社会科学调查中心

二０一八年六月

目录

第一章　项目的基本概述......3
1.1 项目背景概述......3
1.2 项目调查的对象......4
1.3 关键概念解释......5
1.4 问卷生成规则......6
第二章　问卷设计及结构......8
2.1 问卷结构......9
2.2 问卷部分核心概念......10
第三章 调查流程......15
3.1 调查流程......15
3.2 联系代码及其填写方法......18
3.3 样本发放流程......20
第四章 调查员及督导工作要求......21
4.1 访谈准备，原则与技巧......22
4.2 访问中的常见问题与应答......27
4.3 执行督导职责......28
第五章　访问技巧......29
5.1 顺利通过大门......30
5.2 进行预约......36
5.3 建立融洽关系的技巧......36
第六章　数据质量概述......38
6.1 调查员的因素......39
6.2 被访者的因素......42
6.3 调查的环境及时间......46
第七章　附录......49
附录 1：接触登记表......49
附录 2：家庭联系代码......51
附录 3：个人联系代码......52
附录 4：督导确认代码......53
附录 5：调查回执......54
附录 6：招聘流程......55
附录 7：村居介绍信......56
附录 8：家庭预约信......57
附录 9：督导复核问卷......58
附录 10：督导复核报告表......60
附录 11：工作流程......61
附录 12：调查补充说明......62

第一章　项目的基本概述

1.1 项目背景概述

社会科学研究越来越强调量化和实证分析，其特征在于可以将科学知识编码化。其研究的范式是，通过收集和运用系统的调查数据，阐释具有理论或实践意义的议题，而社会调查则是重要的手段之一。基于这个认识，中山大学社会科学调查中心从 2012 年开始推进在中国大陆进行劳动力动态调查项目。劳动力动态调查的目的是，通过对城乡以社区为追踪范围的家庭每两年的追踪调查，系统地监测村/居社区的社会结构和劳动力及其家庭的变化与相互影响，收集和建立中国社会劳动力、家庭和社区这 3 个层次的变动趋势的追踪资料数据库，从而为进行实证导向的高质量的理论研究和政策研究提供基础数据。

CLDS 聚焦于中国劳动力的现状与变迁，内容涵盖教育、工作、迁移、健康、社会参与、经济活动、基层组织等众多研究议题，是一项跨学科的大型追踪调查。为保证样本的全国代表性，CLDS 的样本覆盖了中国 29 个省市（除港澳台、西藏、海南外），调查对象为样本家庭户中的全部劳动力（年龄 15 至 64 岁的家庭成员）。在抽样方法上，采用多阶段、多层次与劳动力规模成比例的概率抽样方法（multistage cluster, stratified, PPS sampling）。在追踪调查方式上，CLDS 在国内率先采用轮换样本追踪方式，既能较好地适应中国剧烈的变迁环境，同时又能兼顾横截面调查和追踪调查的特点。

CLDS 项目于 2011 年 3 月 17 日正式启动；同年 8-9 月， CLDS 以广东为范围开展了试调查，试调查为评估跟踪调查的样本丢失情况，测试问卷等方面积累了调查执行经验；2012 年 6 月，中山大学联合国内 27 所合作院校，启动了在全国范围内的第一期正式调查。项目采取计算机辅助调查技术（Computer Assisted Personal Interviewing, CAPI）开展入户访问，在 800 多名老师、调查督导和访问员的共同努力下，CLDS 项目 2012 年完成对全国范围内 303 个村居，10612 个家庭以及 16253 个劳动力个体的访问。CLDS 项目于 2014 年完成了第一次追踪调查，于 2016 年完成了第二次追踪调查。

2018 年调查将对 2016 年访问的村居，家庭以及个体进行追踪访问。另外，由于采用的轮换样本追踪方式，CLDS2018 有 96 个新的村居进入调查范围，这些村居及其范围内抽选的家庭户将在 2018 年进行第一访问。

CLDS 数据将服务于以下三类人： 1、从事中国社会和劳动力研究的社会科学研究人员和学生；2、将中国社会和劳动力列入研究范围的跨国比较研究人员；3、中国的政策研究人员以及其他有可能使用该数据对中国社会和劳动力进行政策设计和评估的人员。

1.2 项目调查的对象

由于 CLDS 项目采用了轮换追踪方式，2018 年有 96 个新增社区进入调查范围，因此 2018 年的调查对象既有追踪社区，又有新增社区；既有 2018 年访问过的旧家庭，又有 2018 未访问过的新家庭；既有 2018 年访问过的劳动力个体，又有 2018 年未访问过的新个体，具体如下图所示：

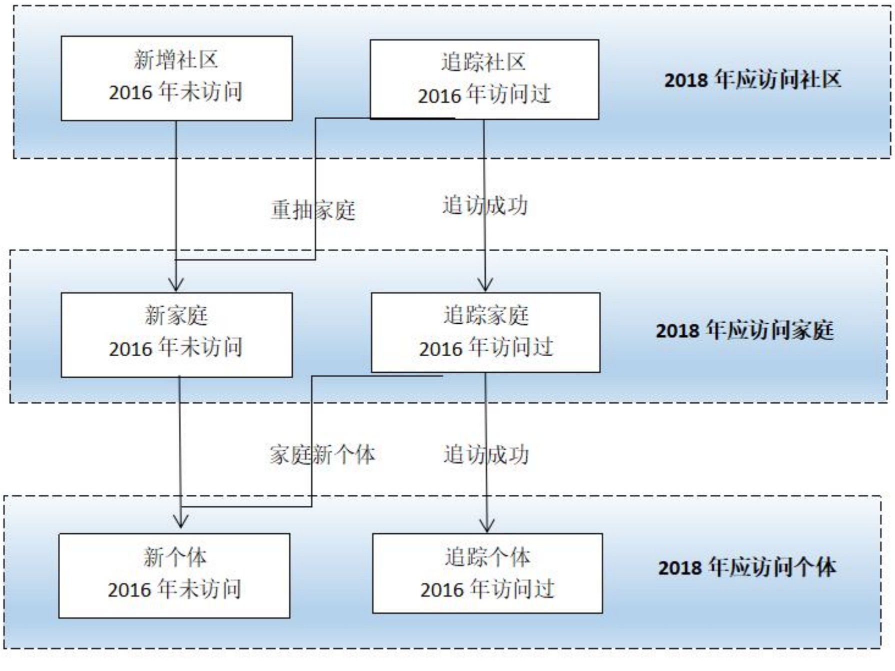

访问的社区是在抽中的县区（PSU）中抽取的，PSU 中所有村居被随机分成 4 份，每一份的全国总体都对全国具有代表性；因此，新增的 96 个社区对全国具有代表性。村居问卷由村居委会负责人接受访问，完成一份村居问卷，主要了解

社区人口，经济，社会组织，基础设施，治安环境等情况。

追踪社区中的家庭，由 2016 年采取地图地址法制作的抽样框中随机抽取，2018 年对这些家庭进行跟踪访问；对于跟踪访问不成功的家庭，为了保证抽取的家庭对该社区具有截面代表性，由 2018 年更新过的样本框中重新抽取家庭进行补充，重新抽取的家庭为 2018 年的新家庭。新增社区中的家庭，是在 2018 年通过地图地址法建立的样本框中随机抽取的，全部都是新增的家庭。

家庭同住成员中所有劳动力个体（15-64 岁）都是个体访问对象，65 岁以上目前有工作的家庭同住成员同样是个体访问对象。农村地区的非同住家庭成员，符合一定的筛选条件，需要由家人代答外出成员问卷。对于追踪家庭，2016 年所有家庭成员为基因成员，基因成员在本社区成立的新家庭，需要对这个家庭进行访问。

1.3 关键概念解释

住宅：住宅指的是我们在样本框中抽取的住宅，有可能是住的是单个的家庭户，也有可能住有多个家庭户。访问员拿到样本，依照样本地址找到相应的住宅，先要询问该住宅住了多少户，如果住有很多户，只有进行了住户抽样，抽中需要调查的家庭户之后，才能开始问卷。但对于追踪家庭，不需要进行住户抽样，只要根据地址，家庭电话等信息找到去年访问过的家庭就行。

家庭成员：CLDS 家庭的边界由被访者自己界定，即他认为哪些人是他的家人，包括同住的家庭成员与非同住的家庭成员。访问员在做家庭过滤问卷之前，先要将我们的家庭概念对他进行说明，并了解一下他家的大致情况。这样能避免接下来做家庭成员过滤时由于双方理解造成的错漏。

同住家庭成员：指的是目前住在调查住宅中的家庭成员，即在家庭成员过滤问卷中问到的“该家庭成员住在家里吗？”，回答“是住在家里”的成员。家庭成员长期住在此处，只是短期出差，探亲访友，外出旅游等，也应算同住家庭成员。另外，住在学校的中小学生也应该算成住在此处的家庭成员。

非同住家庭成员：非同住指的是不住在一块，但被访者认为是一家人的家庭成员。不住在一块包括经济上是一家，但分开居住（例如由子女赡养的父母与结婚的子女分开居住；例如被访者本人是外出打工的，家人留在家乡等）；家庭成

员外出打工，外出上学，出境；家庭成员嫁出，分家；家庭成员参军/服刑，出家，离家出走等等。

同住非家庭成员：指的是目前住在被访问的家庭户中，但不是该家庭的家庭成员的人，比如住家保姆，长工，园丁，长期住在家里的朋友，亲戚等。

基因成员：这是一个只有在追踪家庭才会出现的概念，指的是第一次访问该家庭时的家庭成员。基因成员分家之后，在本社区新成立的家庭，需要进行访问。

主事者：主事者指清楚家庭整个经济状况且在家庭大事决定上起决定性作用的人。只有一个人的家庭，家庭主事者就是被访者本人。

户主：指被访家庭户口登记本上登记的人。如果家庭有多个户主，这里选择最主要的那一个户主填答；如果被访家庭是新成立的，户主户口都待定的，这里暂时将主事者定义为户主。另外，在租房户中，户主不是没有住在此的房东。

家庭 2017 年的收入：这里容易出问题的是那些刚刚组建的家庭，2017 年的收入应该如何计算。我们其实是想侧面了解这个家庭经济状况，如果出现因为家庭刚刚组建 2017 年收入无法计算的问题，请访问员计算被访者家庭 2017 年全年的收入来代替。

1.4 问卷生成规则

考虑到调查成本，调查时间以及重复抽样的问题，CLDS 并不会对所有被访问家庭的家庭成员进行访问。对于同住家庭成员，只要符合劳动力年龄或者超过年龄但在劳动状态，都需要回答个人问卷；对于非同住家庭成员，在农村地区，目前是农业户籍，并且是家庭问卷回答者的晚辈和同辈，并且现住地是在乡镇街道之外的，需要代答外出成员问卷；对于非同住家庭成员，在居委会社区，非流动人口家庭（户主户口与居住地在同市），非同住成员为流动人口的（户籍与居住地不在同一个市的），并且是家庭问卷回答者的晚辈和同辈才需要代答。对于同住的非家庭成员，只有居住满 6 个月，并且符合劳动力年龄或者超过年龄但在劳动状态，才需要回答个人问卷。

离去基因成员，只是针对追踪家庭而言的。家庭基因成员不住在家里，不住在家里的原因为嫁出，离婚或者分家的，属于新组家庭，这些家庭如果还住在本社区，本次需要调查新成立的家庭。CLDS 的基因成员所在家庭，只要仍在被调

查的社区，也是 CLDS 当年的访问对象，不过一旦这些家庭没有基因成员存在（如基因成员从属于新的家庭或者死亡），则终止调查。具体问卷生成规则如下图所示：

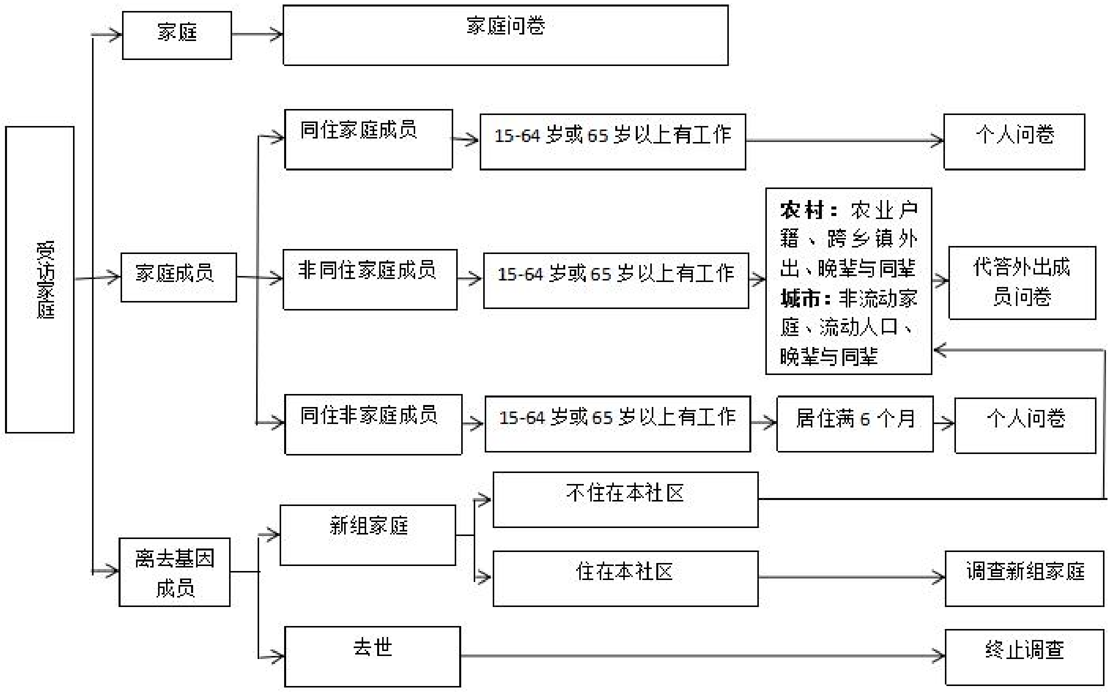

第二章　问卷设计及结构

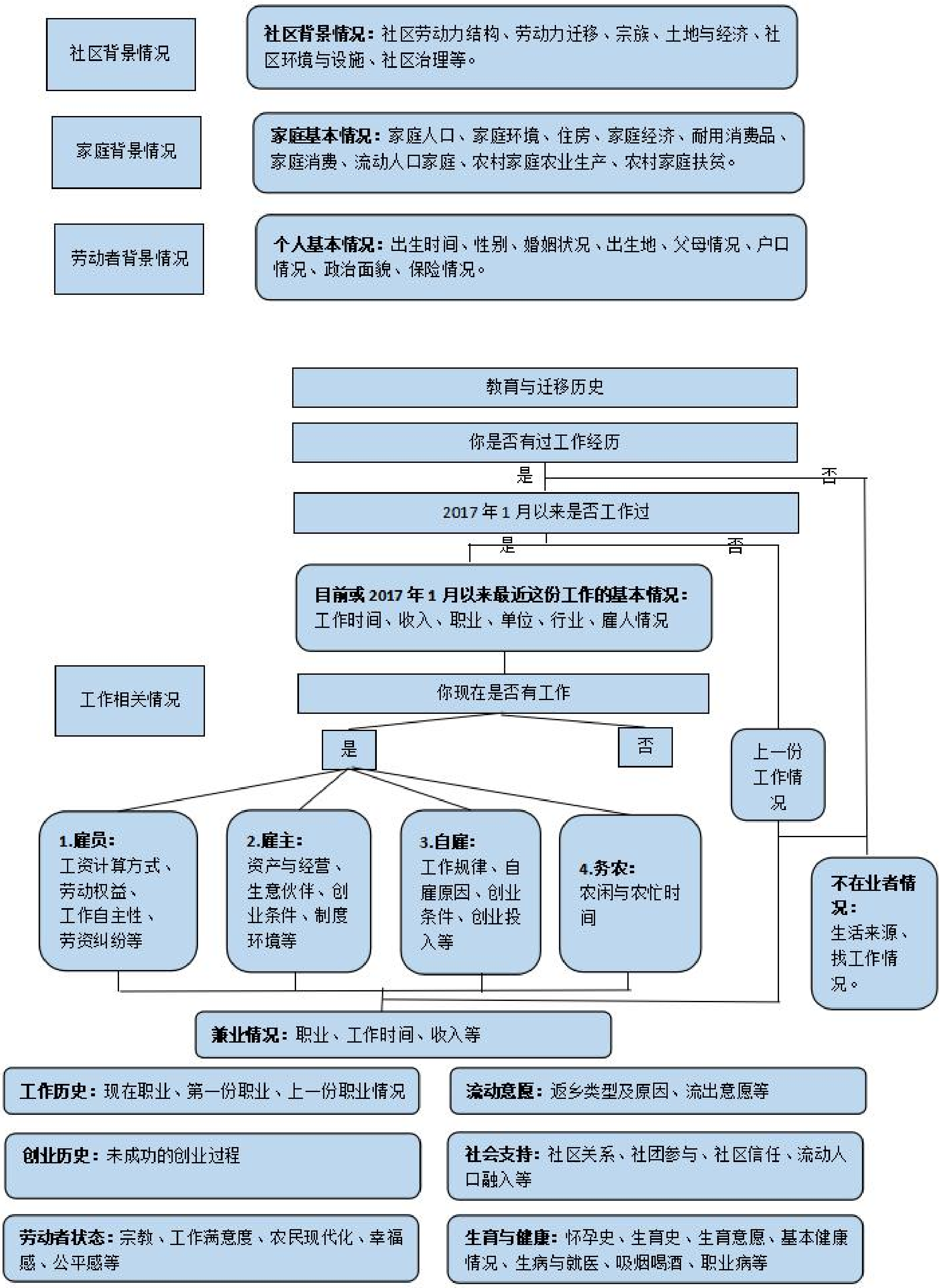

图 1：CLDS2018 年问卷结构

2.1 问卷结构

CLDS2018 围绕劳动力议题，在家庭层面及个人层面上设计收集与劳动力相关的不同信息，如上图 1 所示：

一、劳动者背景情况

家庭情况与个人基本信息作为劳动者基本背景信息收集，CLDS 收集的家庭背景信息包括家庭人口、家庭外出人口、家庭环境、家庭住房、家庭资产、家庭消费、流动家庭与家乡的联系及在当地的融合、农村家庭农业生产情况等。CLDS 收集的个人背景信息包括年龄、性别、婚姻状况、参军、户口、父母情况、保险等信息。

二、教育经历与迁移历史

教育培训与迁移历史放在了一个模块，教育经历详细了解被访者的教育经历，包括所受过的每一阶段教育的开始年份，毕业情况及其学校等级；培训经历主要了解培训的时间、内容以及进一步了解职业资格证书情况。迁移历史询问的是被访者出生地，14 岁的居住地，以及 14 岁到现在所有的跨县市迁移经历。

三、工作相关情况

工作相关情况是问卷的核心部分，在该部分中，工作情况包括有工作和无工作两种情况：有工作主要了解被访者的基本工作情况，包括工作时间、工作收入、具体职业信息等情况。目前有工作的人，按照工作性质分成了雇员、雇主、自雇和务农四部分。雇员部分主要了解工资形式、合同、加班、自主性、福利、管理权、工会、权益受侵犯情况、工作体力要求、工作交往情况。雇主部分主要了解经营的行业、所有权情况、投入资金及渠道、创业社会网络、雇员工作时间、工资、加班情况、生意成本等情况。自雇部分主要了解其工作时间、创业原因、顾客与服务对象、技能要求、工作纠纷等。务农部分主要问了农忙农闲时间。目前没有工作的人，询问了失业原因、期间生活费来源以及找工作意愿，求职情况等。这部分除了收集主要工作情况，还收集了兼职情况，包括兼职的原因，职业，收入，未来计划等。

四、工作史

工作情况部分除了收集当前工作情况，还收集了被访者第一份工作情况，上一份工作情况，以及之前的创业情况。了解原来工作的工作内容，收入，职业级

别，单位性质等情况。

流动意愿是针对农村地区劳动力收集的，在城市化的大趋势下，CLDS 收集了农业劳动力的返乡及其与家乡的联系；收集了农业劳动力的外出工作的意愿。

五、创业过程

创业史询问的是每一个人曾经的创业情况，创业原因以及最后创业的结果等。

六、社会参与与支持

这一部分主要了解被访者的政治参与的和获得社会支持的情况，包括投票情况，获得个人社会关系支持、社会或政府组织支持、社区信任度等情况也包括流动人口居住地的本地人状况、方言水平和返回家乡的意愿情况。

七、劳动者状态

该部分主要了解被访者的民间信仰行为与观念、宗教信仰行为与观念、工作情况的满意度（包括工作收入、工作环境、晋升机会等）、工作价值观、生活满意度、幸福感、信任度、责任感、自评社会地位、公平感、未来工作预期情况。

八、生殖生育

这一部分主要包括怀孕史，生殖人数，生育意愿等情况。

九、健康状况

健康状况主要包括被访者的身高、体重、自评身体状况、过去两周患病情况、健康状况对工作与生活的影响、吸烟与饮酒情况、疾病史、职业伤害、职业病与工伤情况。

2.2 问卷部分核心概念

有过工作经历：工作指从事有收入的活动，务农、兼职、帮忙家庭生意也算在内，但不包括义工、学生兼职、志愿者、家务劳动这些活动。一般一个人只要不是以义工，学生，志愿者等身份赚过钱，就是有过工作经历。

2017 年 1 月以来是否有过工作：参考上面的定义，这里指的是 2015 年 1 月以来从事过有收入的活动，务农、兼职、帮忙家庭生意也算在内，但不包括义工、学生兼职、志愿者、家务劳动这些活动。

目前是否有工作：指的是目前是否正在从事有收入的活动，务农、兼职、帮忙家庭生意也算在内，但不包括义工、学生兼职、志愿者、家务劳动这些活动。

具体职业：此题询问的是被访者目前或者失业前、退休前所从事的具体职业。所谓“职业”，简单地说就是指一个人的工作岗位、工作内容、工种或工作职责，例如“司机”、“售票员”等。请访问员尽可能让被访者描述其工作的具体内容或职责，然后将其记录下来，越细越好。关于具体职业的填写要求如下：

本题被设计为要求被访人描述自己工作内容、工作岗位、工种或工作职责，然后由访问员直接记录，最后在问卷回收后统一根据人口普查的职业编码进行后编码。因此，访问员的细致询问和文字记录是非常重要的。

被访者可能会说得很笼统，如工人、职员、研究人员等，这时访问员应进一步追问：如是工人，那么是做什么的工人，是瓦工、印刷工、钳工、修理工、司机……？如是职员，是从事什么工作的职员，会计、售货员、统计人员、商务调查人员、推销员（推销员中还能分类如食品推销员、医疗器械推销员、人寿保险推销员等）……？如果是秘书，是厂长秘书还是党委秘书，还是厂长办公室秘书……？如是统计人员，是劳动工资统计人员，还是生产统计人员……？如果是管理人员，是公司生产管理人员还是车间生产管理人员，是食堂管理人员还是仓库管理人员……？如果是会计，是公司总会计师还是财务科成本会计……？如是研究人员，是什么学科的研究人员，金融、财会、工程、计算机、哲学等……？如是教师,是大学、中学还是小学教师……，是哪一课教师?

工作单位：工作单位应该是一个独立的机构，有自己的财务和人事管理职权。如果受访者的工作机构分很多层级，无法区分哪一级是自己的单位时，可以提示，受访者工资关系所在的那一级，就可能是他/她的单位：由劳务派遣机构派出的保安，劳务工，家政服务员等，劳务派遣机构是其单位；个体经营者及其雇员的单位应该属于个体工商户。务农与自由工作者（自由职业者，零散工，摊贩，无派遣单位的保姆，自营运司机，手工工匠等）属于无单位的劳动者。

很多受访者对于自己单位的性质可能也不是十分清楚。在这种情况下调查员应确认受访者对于这类问题是否比较清楚，然后将他们的回答记录下来。

题目中“民营”也是一个含混的说法，调查员应该根据问题中的选项，追问受访者回答的“民营”是民营“集体企事业”单位呢还是私营？对于一些实行股份制的单位，现在通行的方法是按照占控股地位的股份的性质来确定单位的所有

制性质，如果是国有股份占控股地位，该单位就可算为国有单位。当然，最终答案应以受访者的确切答复为准，调查员不应根据自己的看法代替受访者的回答。

有的受访者可能会说，他所工作的那个“集体企业”实际上是私人企业。调查员应该询问清楚，受访者是否清楚该单位注册的性质，以此为准记录，受访者本人的猜测不能作为根据。如果被访者对几种单位类型和性质实在不了解和区分不来，访问员可以参考一下定义给予简单的解释。

党、政、军机关：是军队、武警、民兵和中国共产党的各级组织、部门、机关和各级人民政府所属的部门、机关以及其他民主党派、社会团体、部分被授有一定行政权力的事业单位的总称。

国有企业，或称国营企业：国际惯例中，国有企业仅指一个国家的中央政府或联邦政府投资或参与控制的企业；而在中国，国有企业还包括由地方政府投资参与控制的企业。

国有、集体事业单位：是指国家、集体投资兴办、管理从事科研、教育、文化、体育、卫生、新闻、广播电视、出版等单位。

集体企业：是指财产属于劳动群众集体所有、实行共同劳动、在分配方式上以按劳分配为主体的社会主义经济组织。集体企业按举办的主体可以分为城镇集体企业和乡村集体企业。

个体经营：是生产资料归个人所有，以个人劳动为基础，劳动所得归劳动者个人所有的一种经营形式。个体经营有个体工商户和个人合伙两种形式。

民营，私营企业：是指生产资料属于私人所有，雇工 8 人以上的营利性的经济组织。

外资/合资企业：指外商独资或中外合资企业，即外国的公司、企业、其他经济组织或者个人，依照中国法律在中国境内设立的全部或部分资本由外国投资者投资的企业。

民办非企业、社团等社会组织：是指企业事业单位、社会团体和其他社会力量以及公民个人，利用非国有资产举办的从事非营利性社会服务活动的社会组织。如各类民办学校、医院、文艺团体、科研院所、体育场馆、职业培训中心、福利院、人才交流中心等。社团又称社会团体，指中国公民自愿组成，为实现会员共同意愿，按照其章程开展活动的非营利性社会组织，主要是指以文化、学术或公益性为主。成立社会团体必须经其业务主管单位审查同意，必须同时接受登

记管理机关（民政）、业务主管单位的监督。

单位规模：指被访者所在单位的员工人数，这里的规模与工作单位的界定是相联系的，被访者工作单位确定好了，才能估计出单位的规模，特别是在被访者单位层级特别多的时候。

雇员：（即被雇佣者），是指被雇请的任何个人，如工人被雇请做各种杂活，法律顾问、律师、会计、摄像师等其他类别的劳工等。雇员分为很多阶层，如蓝领和白领，企业的管理层和普通工人，政府高官和普通公务人员，大学教授和普通助教，餐厅有经理和服务员（侍者）等等。在国家党政机关、人民团体、军队；国有或集体事业单位；国营企业或集体企业，村居委会工作的人员，无论职位高低，都属于受雇于国家的雇员。

雇主：在中文术语里面指老板，指雇请被雇佣者（劳工）的实体或者个人，雇佣者通过支付薪水以交换劳动者所付出的劳动。雇佣者拥有支配权，拥有土地、资本，同时也是知识产权的拥有者。CLDS 的界定中，雇主指的是雇佣 1 人及以上的个人，其中雇佣指的是给人定期发工资。

自雇：或称自雇人士，其工作的雇主就是自己。其工作的雇主就是自己，同时也不雇佣他人工作。在 CLDS 调查中，自雇包括各类没有雇佣人的个体店主（包括额没有申报营业牌照的个体店主）。还包括自由工作者（自由职业者，零散工，摊贩，无派遣单位的保姆，自营运司机，手工工匠等）。

务农：指直接从事种植业、林业、牧业、副业、和渔业**生产**的职业（如种地、养殖鸡鸭水产等）。

户口迁移：户口迁移指的是被访者的户口登记地从一个地方迁到另一个地方。

创业：指商业行为者在一定的创业环境中识别并利用机会、动员资源、创建新组织，发起、维持和开展以利润为导向的有目的业务活动。比如开商店，开公司，做生意等。

兼职：兼职指的是劳动者指在不脱离原组织的情况下，利用业余时间从事第二职业并取得一定的报酬。兼职者除了可以获得本职工作的收入外，还可以按标准获取所兼任工作的其他收入。兼职包括网上兼职和现实生活中兼职。

流动人口：本次 CLDS 的流动人口参考了人口普查的定义，户口所在地在本乡镇街道以外的人口都是流动人口。不过流动人口不应该包括市区内人户分离的

人口，所以问卷部分专门针对流动人口的问题，属于市区内人户分离的人，系统也会跳出这部分题目需要回答，这时候可以填答 99998（不适用）。

迁移经历：指从一个县市迁移到另一个县市，并连续居住六个月及以上；同一个城市内区与区之间的迁移不算，同一城市内县与区之间的迁移算；如果被访者目前到本地居住不满半年，也算一次迁移经历。

工资性收入:指就业人员通过各种途径得到的全部劳动报酬，包括所从事的主要职业的工资以及从事第二职业、其他兼职和零星劳动得到的其它劳动收入。它包括六个部分：

1、计时工资：计时工资是指按计时工资标准（包括地区生活费补贴）和工作时间支付给个人的劳动报酬。包括：对已做工作按计时工资标准支付的工资；实行结构工资制的单位支付给职工的基础工资和职务（岗位）工资；新参加工作职工的见习工资（学徒的生活费）；运动员体育津贴。

2、计件工资：计件工资是指对已做工作按计件单价支付的劳动报酬。包括：实行超额累进计件、直接无限计件、限额计件、超定额计件等工资制,按劳动部门或主管部门批准的定额和计件单价支付给个人的工资;按工会任务包干方法支付给个人的工资；按营业额提成或利润提成办法支付给个人的工资。

3、奖金：奖金是指支付给职工的超额劳动报酬和增收节支的劳动报酬。包括：生产奖；节约奖；劳动竞赛奖；机关、事业单位的奖励工资；其他奖金。

4、津贴和补贴：津贴和补贴是指为了补偿职工特殊或额外的劳动消耗和因其他特殊原因支付给职工的津贴，以及为了保证职工工资水平不受物价影响支付给职工的物价补贴。 津贴包括：补偿职工特殊或额外劳动消耗的津贴,保健性津贴,技术性津贴,及其他津贴。物价补贴包括：为保证职工工资水平不受物价上涨或变动影响而支付的各种补贴。

5、加班加点工资：加班加点工资是指按规定支付的加班工资和加点工资。

6、特殊情况下支付的工资：特殊情况下支付的工资包括：根据国家法律、法规和政策规定，因病、工伤、产假、计划生育假、婚丧假、事假、探亲假、定期休假、停工学习、执行国家或社会义务等原因按计时工资标准或计时工资标准的一定比例支付的工资；附加工资、保留工资。

加班定义：国家实行劳动者每日工作时间不超过 8 小时、平均每周工作时间不超过 44 小时的工时制度。在法定休假日，用人单位应当依法安排劳动者休假。

不符合这三个定义中的任何一个，都算加班。

工伤定义：在工作时间和工作场所内，因工作原因受到事故伤害；工作时间前后在工作场所内，从事与工作有关的预备性或者收尾性工作受到事故伤害；在工作时间和工作场所内，因履行工作职责受到暴力等意外伤害；因工外出期间，由于工作原因受到伤害或者发生事故下落不明；在上下班途中，受到非本人主要责任的交通事故或者城市轨道交通、客运轮渡、火车事故伤害；其他经认定为工伤的情形。

第三章　调查流程

3.1 调查流程

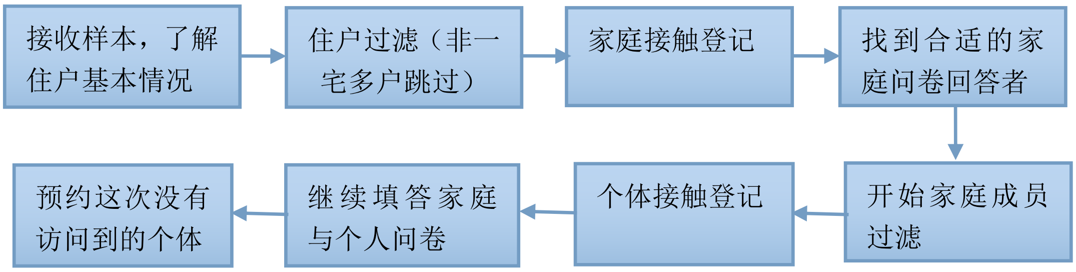

图 2：调查员调查流程图

一．接收样本

所有的样本由调查中心发给中大督导，由中大督导发给当地执行督导，执行督导直接发给访问员。

二．联系村居委会工作人员或被访家户，了解住户基本情况

2.1 根据样本地址及家庭信息，通过村居委会工作人员的帮助或者自己上门，联系到住户，不要马上开始家庭成员过滤问卷，应该首先了解住户基本情况（住了几户，家里住了多少人，多少家人住在外面），传达 CLDS 的“家庭”概念（哪

些人属于家人由被访者自己界定，包括在外的家人）

2.2 填写联系记录。

2.3 如果多次联系不上，空户，多次拒访等特殊情况，将情况汇报给督导，执行督导确认后上报，下发新样本。但是由于调查过程中遇到拒访或者住户暂时不在家的情况很普遍，为了保证调查样本不出现选择性误差，我们提倡不轻易放弃样本，对比较难访或难以沟通的住户进行沟通，必要时可以求助于村/居委会，邻居，门卫等多种渠道；暂时没人在家的住户，我们规定至少上门 3 次，每次间隔至少 6 小时。

三. 开始住户过滤

这个过程是针对一宅多户的情况，比如一个宅子里面住了多户人或者住宅里面住的是集体户，这些情况需要住户过滤，大部分我们发下去的样本地址应该只住有一户人，可以跳过此步骤。即从多个住户中随机抽选一户作为我们的调查对象。因此，访问员在做这一步之前，一定要了解住户信息。

集体户，即无家庭关系的多人共居（如企业员工合租的宿舍、工棚），则以单个人为一户进行记录。家庭户和集体户混合居住在一个地址户内，则家庭户为单独一户，集体户则以每个人为单独一户的方式记录。给每户一个称谓，按开门答话人回答的顺序进行记录。如果开门答话的人不知道住在一个宅子里的其他人姓名或称谓，则用其他方便辨识的信息指代称谓，比如左边第一户，或者房间 101 等。

另外，住户抽样只能抽一次，抽取的家庭即为该地址调查的家庭户。不能因为抽到的那一户难访问而再次进行一宅多户抽样。

四. 录入入户接触情况登记表

每联系一次被调查户，都需要打开手机 APP 填写入户接触情况，若入户成功，则继续以下步骤，若入户不成功，则继续下一户的入户工作。

五. 找到合适的家庭问卷回答者

家庭问卷尽量找户主或主事者，或者对家里情况了解比较多的人来回答；尽

量找稳定的家庭成员来回答家庭问卷，不要找即将嫁出或分家的儿女。

六（A）．开始家庭成员过滤：（新增家庭）

5.1 除了您之外，您的家人还有多少人，每一个人的情况（家人为被访者自己界定的，包括在外的家人）

5.2 除了这些人还有其他家人吗？（家人为被访者自己界定的，包括在外的家人）

5.3 除了家人外，目前家里还住了哪些非家庭成员

5.4 农村地区家庭外出成员筛选：确认是否 15-64 岁；确认是否是家庭问卷回答人的晚辈；询问是否是跨乡镇街道，即在本乡镇街道外。

六（B）．开始家庭成员过滤：（追访家庭）

5.1 下列家庭成员，信息是否准确，纠正。

5.2 除了以上这些家人，您的家人还有多少人，每一个人的情况（家人为被访者自己界定的，包括在外的家人）

5.3 除了这些人还有其他家人吗？（家人为被访者自己界定的，包括在外的家人）

5.4 除了家人外，目前家里还住了哪些非家庭成员

5.5 农村地区家庭外出成员筛选：确认是否 15-64 岁；确认是否是家庭问卷回答人的晚辈；询问是否是跨乡镇街道，即在本乡镇街道外。

七. 录入个体接触情况

在每开始每一个个体问卷访问之前，先填写个体接触情况，若成功，则继续如下步骤，若不成功，则继续接触下一个个体。

八. 继续家庭问卷或者开始个体问卷

完成过滤问卷之后，该访问员继续询问家庭问卷问题，而该家庭应做的个体问卷都出来了（没有则是这个家庭没有合适的劳动力，不需要做个体问卷）。这时候如果是多人入户做调查，可以开 wifi 共享，另一个访问员链接到主做访问员的电脑，输入个体代码，开始个体的访问。

九．预约没访问到的个体

家庭内的劳动力个体，经常无法一次性做完，比如有人上班去了没有回家，比如将要做饭没有时间等，这些劳动力个体，应该进行预约，预约合适的时间再次上门访问，直到将家庭内所有个体问卷全部做完。

3.2 联系代码及其填写方法

联系代码是访问员将每次对家庭或家庭劳动力个体进行联系时的联系情况的记录，具体联系代码参见附录 2。联系代码的填写是访问员访问流程中的重要步骤，访问员一定要将联系代码详细的认真的记录下来，因为中大督导将在系统中根据历次联系情况分配样本最终代码。如果访问员联系代码填写不全或者备注不够清晰，将会影响确认代码的发放，耽误调查进度。

1.联系代码简介

家庭联系代码：

100（开始问卷）：指访问员联系这个家户，家庭马上答应开始问卷了，这时候应该选择此联系代码；

110（上次因故中断，此次从上次中断处开始）：如果被访者上次有事中断了调查，约了这次开始，这次上门开始问卷，应该选择此联系代码；

120（中途拒访）：如果家庭问卷被访者答了部分家庭问卷信息之后，不愿意回答了，多次说服都没有结果，这时候访问员退出之后，再次点击这个样本，应该填上此代码。

210（没有联系上）：指没有接触到此家户被访者，包括情况很多，比如敲门没有人应答，无法进入建筑物/无法进入小区，打电话没人接等；需要注意的是，2106（受访户不住在此处，住在此村居）；2107（受访户不住在此处，不住在此村居）以及 2108（受访户不住在此处，不确定地址）这 3 个代码是针对追踪家庭才会有的，只有追踪家庭我们才追踪家户而不是地址；而新增家庭，我们是样本框本身是地址，只要样本地址中住了家户，都能成为访问对象。

220（联系到被访家庭，没有拒绝）：指的是访问员已经和被访问家庭取得了联系，被访问家庭没有拒绝，但是无法马上开始调查，需要再约时间的情况。

230（联系到被访家庭，拒访）：指的是访问员和被访问家庭取得了联系，但家庭拒绝接受调查的情况。

320（受访家庭不便接受调查）：指的是受访问的家庭，由于婚丧嫁娶或者照顾病人等特殊原因不便接受调查的情况。实际调查过程中，要尽量减少这种情况的发生，访问员遇到这种情况，需要与当地村居委会及执行督导沟通决定如何处理。

331（空户）：指的是访问员已经确定样本地址是没有人住的情况，这种情况要在备注中将判断依据写明。空户要和多次联系联系不上的情况区分开来，后者指的是家庭住址中有人住，但是联系的时候没有人。前者指该地址确实没人居住。

332（非住宅）：这种情况指的是样本地址不是一个居住的住宅，比方说是一个仓库或一个旅馆等。

333（在建）：指的是样本地址的房屋目前正在建设中，没人居住的情况。这种情况可能在农村地区比较多。需要在备注中说明情况。

340（无法找到绘图地址）：绘图地址指的是有些样本地址写的不是具体地址，而是根据我们的绘图手册建筑物编号，楼层及楼层右手边数第几户的规则编写的地址。访问员按照这个地址找不到相应的家户，比方说样本地址说第六层右手边数第 5 户，而实际上第六层只有 4 户人，没有第 5 户。这种情况的出现与绘图及核查地址不仔细有关，需要访问员将情况具体记录下来。

个体联系代码：个体联系代码基本上与家庭联系代码类似，不过少了 331、332、333、340 等与样本地址相关的代码。

2.联系代码的填写

每个样本点击开始问卷之后，马上有一个样本的状态需要提交，并且有联系方式可以选择，还有备注信息可以填写，如下图 3 所示：系统自动生成了日期/时间，这个日期/时间可以还可以修改。每一次联系，最好都能打开手机，将联系情况记录下来。

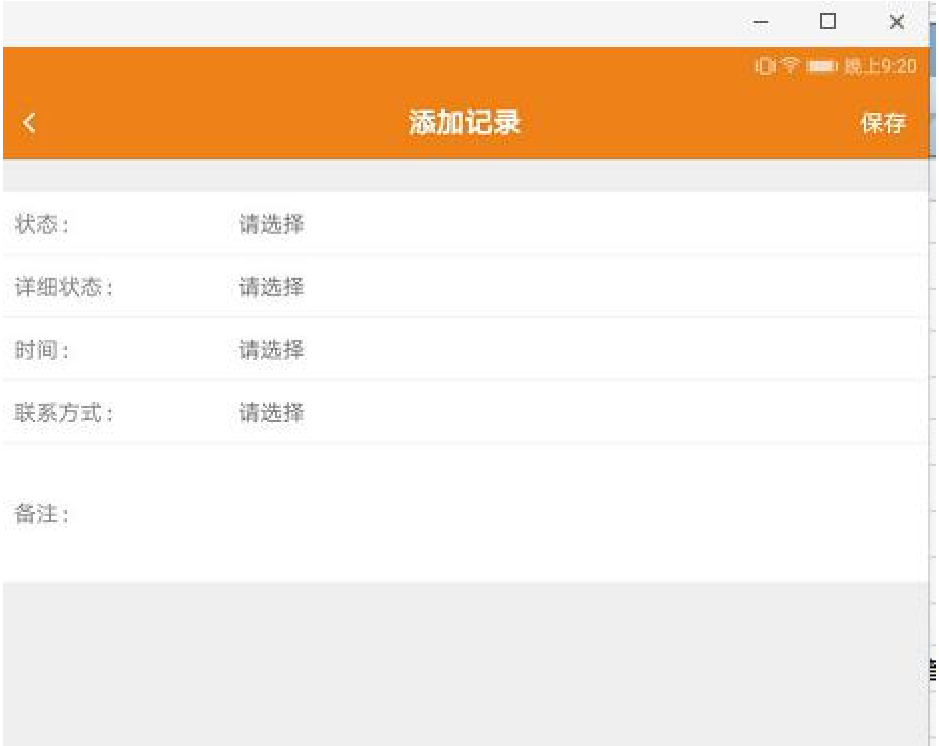

图 3：联系代码填写界面

访问员需要注意的是，联系代码填写前面说的两个地方都得填写齐全。最开始的系统填写界面处需要补齐并且说明情况，因为中大督导是根据这里记录的联系代码进行确认代码的发放的，没有填写齐全就会影响到新样本的发放，拖慢调查进度。

3.3 样本发放流程

各地将绘图资料发送回中山大学社会科学调查中心后，中山大学社会科学调查中心的抽样工作人员马上对绘图资料进行审核，审核完成之后将备选地址导入系统进行抽样及样本发放。

新增的社区会随机抽取 35 户发送到中大督导，中大督导将这 35 户分配给相应的执行督导，执行督导再将 35 个样本按照一定原则（如工作量、地理位置）分配给相应的访问员。追踪社区首先给的 35 户就是 2016 年做过的 35 户，不需要随机抽取。

当这 35 户出现拒访，多次联系不上等访问不成功的情况时，访问员需要将多次联系情况补充完整，该写备注的地方写清楚备注，将数据发送，然后将要换

样本的情况告知执行督导。当一个村居要换样本的数量达到 5 个或以上（当然，如果整个村居只剩下少于 5 个样本未完成是例外），执行督导可以向中大督导申请补充分发样本（即：换户）。每个样本的情况是什么，执行督导需要清楚并告知中大督导。访问员数据发送成功之后，中大督导登录系统，就能根据访问员联系情况以及执行督导的反馈确认最终代码，并向中山大学社会科学调查中心申请换户。换户时，请说明需要换户的是哪个社区，换多少个地址。

发放样本的工作比较重要，涉及到实地执行的进度、效率和质量管理。要将样本发放工作做好，需要访问员，执行督导，中大督导以及中山大学社会科学调查中心抽样工作人员全力合作：

第一步：访问员需要按照规定，最好该做的事情，比如只有多次无应答的，才申请换户；比如拒访的样本写清楚备注；比如数据一定要发送等。

第二步：执行督导需要了解样本使用情况，及时将情况告知中大督导。如果中大督导根据联系情况记录对有些样本有疑问而无法分配最终代码时，执行督导需要立即向访问员了解实际情况，并将情况告知中大督导。

第三步：中大督导需要及时响应换户需求，正确做好确认代码。

第四步：中山大学社会科学调查中心工作人员，需要及时做好抽样并将样本及时分配下去。

（此部分详见《绘图抽样手册》中的第十三章“样本发放流程”）

第四章 调查员及督导工作要求

调查员是数据的收集者，直接面对调查对象收集信息。在调查期间，调查员的态度、兴趣、参与和合作是非常重要的。

（1）每一位调查员都应当努力去争取调查的成功，并且应当每天都坚守工作岗位。（2）调查员如果有事不能按时参加培训和实地调查，必须事先向小组带队老师或督导员说明情况或请假。（3）为了做好这次调查，时间和日程安排是非常严格的，所以调查员必须按照日常安排准时参加培训课程，正式调查启动后必须按照计划准时开始外出调查。（4）每一个调查小组在工作中应该紧密合作，分担困难，相互支持。应当在调查小组中形成友爱团结的气氛。（5）调查

员必须保证调查所收集的数据资料的精确和完整。为达到这一目标，小组带队老师或督导员将随机抽选部分样本，进行核查。（6）调查员必须对受访者在调查中提供的资料严格保密，不应与任何人进行讨论，包括其他调查员。

4.1 访谈准备，原则与技巧

在问卷调查中，有一些共同的基本原则是调查员必须遵守的，这些基本原则对保证调查成功非常重要。

1.善于交往、善解人意

调查员应该是这样一种人：首先，无论受访者的性别、年龄、家庭背景或政治观点，他应该都乐于并善于与之交往、交谈。其次，他对任何人都要富有同情心，善解人意，彬彬有礼，平易近人。记住，调查员不是在进行犯罪调查，也不是进行考试和智力测验。

2.做好调查前的准备

（1）熟悉问卷和调查员手册

在进行入户访问之前，调查员对问卷上的所有问题都应仔细研究明白，充分理解了其中的含义，以应付受访者提出的难题或反驳，必要时对问卷上问题的含义进行解答。这样在访问中才能够做到从容、自信、对可能的疑问给予适当解答，保证访问顺利进行。因此，调查员应不断重温问卷、调查员手册及其他提示，如发现有不理解之处，及时解决。

熟悉问卷和调查员手册对于顺利进行访问是非常重要的。请想一想，当调查员经过努力使得受访者同意接受访问，而且抽出专门的时间接待调查员，这时，如果调查员对于所询问的问题表现得非常陌生，提问时结结巴巴、断断续续，对一些疑问无法给予及时的解决，会使受访者感觉你本身缺乏专业资格，对想要了解的问题完全没有理解，因而对调查员产生不信任、甚至看不起的想法，这会极大影响访问过程的顺利进行。同时，调查员如果比较熟悉问卷，会使得整个访问的过程非常流畅，大大缩短访问时间。

调查员不仅应该熟悉问卷，而且需要掌握一定的技巧。如果仔细分析问卷，我们会发现问卷中有些问题虽然是必须记录的、但不一定是必须当面询问的，例

如，受访者的性别。当受访者就坐在你面前接受访问时，调查员再询问受访者的性别，会使自己显得不够聪明。

（2）安排好一天的工作计划

事先计划好一天的工作时间安排,可以节省很多时间,并保证顺利完成调查，例如如何乘车？如果预定的受访者不在的话，下一个受访者应该是谁？

（3）带好所有的必用品：预约信，电脑，绘图资料，交通图，礼金、手表、空白纸等物品。

3.与受访者建立融洽的关系

调查员和受访者彼此之间起初是陌生的，所以调查员的首要任务之一，就是与受访者建立起一种融洽和谐的关系。受访者对你的第一印象将影响他们对调查合作的态度。因此，你必须穿戴整齐，自我介绍要彬彬有礼。当调查员在某地开始调查之前，小组带队老师、督导员或抽样员应尽可能与当地居委会负责人取得联系，请他们事先告知受访者调查一事。整个调查期间，你也将会随身携带一份“介绍信”，证明你的身份。

（1）建立良好的第一印象

当你初次接触受访者时，应当尽力使对方感到轻松自然，保持一种非正式的交谈方式，不要过于严肃。首先要向受访者友好地表示问候，说明你是中山大学社会科学调查中心委托的一名调查员，并说明来访的目的。然后，告知对方他所提供的一切信息都将会保密。谈话中所使用的语言应简明易懂。如必要，向他们出示“介绍信”。调查员要做到受到受访者的信赖。

（2）态度要不亢不卑

调查员决不要在开始时就以一种歉疚或谦卑的方式向受访者提问，比如“您现在一定很忙吧？”，“您愿意抽出一点时间吗？”，或“您不介意回答一些问题吧？”等等。如果你向受访者这样提问题，很可能调查没开始就遭到拒绝。你应当这样开始：“我想问您一些问题”，或“我想请您谈谈您的家庭情况和职业情况”。

（3）必要时再次强调受访者的信息将受严格保密

如果受访者对接受访问犹豫不决，或对访问后的资料用途有疑问，你就需要向他解释：访问所收集的资料将受到严格保密，个人的姓名绝不会用于任何用途，

因为所有的调查信息只用于综合的统计分析和撰写研究报告。特别要注意，决不能当着受访者的面，与其他调查员谈论已经做过的访问。

（4）坦诚礼貌地回答受访者提出的所有问题

受访者在同意接受访问之前，可能会问你一些关于调查本身以及他们是如何被选中的等问题。你回答这些问题时应当直率和友好。受访者还可能会关心访问将持续多长时间。假如你第一次访问时，受访者难以抽出时间，你就应说明你很乐意再次来访，并约定好下次来访的时间。

（5）单独进行访问

如果访问时有其他人在场，你就有可能得不到受访者真实的答案。所以，你应尽可能对受访者进行单独的访问，以避免小孩儿或从外面进来的人（如邻居）的干扰。因此，在正式访问之前，你应询问哪里是与受访者单独谈话的最好地方。

4.在访谈中保持客观中立的立场

（1）在整个访谈过程中保持中立

大多数受访者一般会是热情好客的，并且在你提问时有可能会迎合你，给出他们认为你所想要的答案。因此，必须特别注意的是，在你提问时，一定要时刻保持绝对的中立，决不要给受访者任何暗示，决不要在你的面部表情或语言中，流露出某种表情或语调，使受访者以为他们的回答是“正确的”或“错误的”。不要对受访者的所有回答，用语言、行动、手势等等流露出惊讶、高兴、反对等情绪。

问卷上的提问都经过仔细斟酌，没有暗示某一个答案比另一个答案更好更合理。如果你不能按照问卷上的问题正确地进行提问，那么你就可能破坏提问中立的原则。假如你问：“您家里有收音机吗？”这是一个中立的问题。相反，假如你问：“您有收音机，不是吗？”就表明你很愿意得到“是”这样的答案。这就是我们所说的“引导性提问”。

不要提出一些引导性的问题。例如，这样的问法是错误的：“您至今没有找到工作的主要原因是什么？是因为您是一位家庭妇女吗？”第一问句是中立的，但在第二句中调查员加入了他自己的“引导性问题”。即使这位受访者没有找到工作是主要是因为其他原因，但出于礼貌或受你提问的影响，她也可能会随声附和：“对，是因为我是家庭妇女”。这就是我们为什么强调要严格按照问卷上的

问法进行提问的原因所在。

说话的语速一定要适中，语言要清晰，特别是当语言沟通比较困难的情况下更是如此。假如受访者的答案模糊或不清晰，为了澄清答案，往往需要提出进一步的探寻性问题，这时仍要保持中立的立场，例如："您能解释得更多一些吗？"，"我刚才没听清楚，请您再重复一遍"，"不要着急，请您再好好想一想。"

即使受访者的答案与某一个提问无关，也不要给以提示，如问"您指的是这个吗？"因为在很多情况下，尽管他们有时所指不是这个，也将会同意你对答案所作的解释。你应当以某种方式，使受访者能够做出与问卷提问有关的回答。

（2）不要随便改变问卷上提问的词和句子

调查员的提问方式应是完全客观的，你的工作是准确记录来自受访者的信息，而不是对所获信息施加个人影响。因此，提问应按照问卷上原有的词句方式，不应当随意改变。如果受访者不理解，就应当逐字逐句清晰地念出来。如果受访者仍然不明白，你可以用其他的字或词替代原来的问句，但小心千万不要改变原来问句的意思。如果你必须对某些问题给予解释、但你又不能完全确定你的解释是否正确时，你应当及时查阅调查员手册。在一般情况下，你应该尽量不要提供提问之外的额外信息，如有必要也应越少越好，以便获得来自受访者的准确答案。

（3）不要推测受访者的回答

有时人们往往会根据受访者的收入、教育水平、家庭背景，推测他们应当怎样回答。但这万万要不得。你应当专注于倾听受访者的回答，而不是你自己猜测！不要事先将受访者设想为某种类型的人。例如，不要把那些从农村来的、或较为贫穷、文化水平较低的人，事先假想为他们一定会有某种工作经历或有某种看法。绝不要假想受访者会说出什么，或向对方提出你的建议。应当严格按照问卷上的提示和跳答类型进行访谈。不要以一种令人难堪的方式提问，比如说"虽然这可能是一个愚蠢的问题，对您不合适，但我还是要问一下"。这样会让受访者感到尴尬和为难。

5.机智地面对不愿配合的受访者

调查中会遇到这样一些情况，有些受访者可能冷淡地说："我不知道"，或给出一些无关的回答，或表现出不耐烦和冷漠的样子，或推翻他们以前说过的话，或拒绝回答。在这种情况下，你必须尽量在谈话中激起他们的兴趣，使他们明白调查只涉及到他们的工作、单位和收入等情况，他们的回答对制定政策和科学研

究是很重要的，不会给他们个人带来任何麻烦。

假如受访者所给出的是一个无关的回答，不要粗鲁地打断他。应该首先听完他说什么，然后有礼貌地引导他们回到最初的问题上。在访问过程中应尽量创造一种良好的氛围，让受访者感到你是一个友善的、善解人意的和值得交谈的人，可以对你说出任何事而不会感到害羞或尴尬。

如果受访者不愿回答某个问题，要想方设法礼貌地转变对方的态度。例如，向他解释本次调查对所有人都是这么问的，并且所有的调查将被综合到一起进行分析。如果受访者仍然拒绝回答，那么就在这个问题旁注明“拒答”，然后就接着问下面的问题。如果你成功地完成了访谈，在结束之前可以机智地对前面未答的问题再问一遍。如果受访者仍然拒绝合作，事后你应向督导员进行汇报。

6. 控制节奏

（1）访谈切忌急躁

提问应当注意节奏，以确保受访者能够理解你所问的问题是什么意思。每问完一个问题，应当有一个停顿，以便让受访者有足够的时间进行思考。如果受访者没有机会仔细考虑，他们就可能会说“不知道”或随便给一个回答。由于我们这次的调查主要涉及受访者的工作状况、工作经历、受教育经历、社会关系和一些态度，可能会需要受访者进行回忆或思考，因此，让受访者有时间充分考虑是非常重要的。

（2）不要失去对访谈过程的控制，防止谈话漫无边际。

7. 衣着庄重整洁，不要穿着奇装异服。

访问员每天需要在调查点来回步行，所以应以穿运动鞋为主。为显示对被访者和其他打交道对象的尊重，穿着避免过于随便，如短裤、拖鞋等。女访问员为安全起见，穿着宽松为宜。

8. 访问中使用的语言

这份问卷对大多数受访者来说没有任何问题。但是，由于调查涉及不同的地区，如果调查员遇到有些人只会说他们的家乡话，自己可能听不懂，那么，调查员应先谢谢这位受访者，告诉他说你会以后再来拜访。然后与带队老师和督导员联系，或者安排另一位调查员，或者找一位翻译。但翻译一般没有受到严格的调查培训，所以，你必须与他密切合作并指导他进行调查。

另外，问卷是用标准的书面语言设计的，个别词汇与当地方言可能回有一些差异。如果你用不同于问卷的语言与受访者交谈，那么，一定要注意不要改变问卷中的原意。

9.调查员的自我保护

大部分受访者是善良友好的，你不会遇到个人安全方面的问题。但遵守下列建议，也许对更好地保护自己是有帮助的：

＊ 不要携带过多的钱或贵重物品。

＊ 穿着庄重，不要过于显眼。

＊ 小心保管你的身份证或其他身份证明。

＊ 如果你感到必要，可带一位伙伴一同前往，但访谈中他（她）应当不影响调查。

＊ 如果受访者或调查员觉得有必要，访谈也可以安排在公共场所或其他比较“公共”地方。

＊ 要表现出镇静老练、训练有素

＊ 如果你觉察出你正处于危险之中时，应立即停止访谈，并且离开！告诉受访者你或你的带队老师或督导员将以后再次来访。

4.2 访问中的常见问题与应答

1、调查有意义吗?

应答：当然有意义！我们开展这些调查，了解民众的生活状况和对社会的看法，就是为了给政府制定社会政策提供帮助的。好的政策，人人都会受益的。比如，制定了好的医保政策，越来越多的人看得起病了……，你的意见是对我们工作的帮助，您是在做一件好事呢！

2、怎么找到了我家？

应答：我们是采用很严格的抽样方法来选择受访人的。你的家庭是我们通过居委会提供的名册抽选。在全国只调查 15000 家，在你们居委会我只调查 35 家，您家刚好就选中了。所以您家就是全国公众的代表，您的意见是很重要的。另外请您放心，我们会根据《统计法》对您的个人信息严格保密。我们最后的调查结果是和全国几万名和您一样的公众的信息汇总在一起的，不会单独公布您个人的回

答的。

3、你说的这些问题我都不懂，你找别人去吧。

应答：您可以自由发表意见，没有对错之分。大家的意见，很难有完全一致的。问卷中的问题，也没有对错之分，你怎么想就怎么回答。如果题目您有不明白的地方，可以告诉我。

4、我很忙，没工夫和你谈，你找别人去吧。

应答：按照我们调查的要求，是不能随便更换受访者的。请您尽量安排时间。我们调查大概需要____小时，您看今天或明天什么时候我再来拜访您？我们从XX来一趟也不容易，麻烦您能留一个电话吗？来之前我和您联系好。这是我的电话号码________________。

5、给我有什么好处呢？

应答：不好意思，第一次上门拜访您，也耽误了您的时间，我们的调查会给您一些报酬，是____元，访问结束后，我会给您的，谢谢！

4.3 执行督导职责

在整个实地调查过程中，小组带队老师或督导员通过对调查的组织、管理和信息反馈，对调查员进行进一步的培训，具有十分重要的作用。我们说的执行督导，是调查队伍中直接面对访问员，管理访问员的带队老师或督导员，一个省市的全部调查队伍中，由于队伍安排或社区数量的差异，可能有很多个执行督导。比如2016年广东省60个村居的调查队伍中，有一两个总督导，18个执行督导。

执行督导应该做到以下几点：

（1）与调查地的联系人、村委或居委联系（携带工作函，见附录五），与被调查社区建立良好的关系。

（2）通过村/居委会对社区住户进行调查前的告知和宣传，在社区公告栏张贴海报；要求做到村居委会带访问员入户。

（3）对调查员提供建议和指导，以便使调查的程序更合理、提问的方式更规范、对问卷中问题的解释更准确。

（4）每天接收由中大督导分发的样本，然后根据访问员的任务及进度，分发给本队的访问员。

（5）每天了解本队访问员分发样本的家庭及个体问卷完成情况，联系情况，出现需要最终确认的家庭或个体，及时向中大督导联系，反馈最终情况，跟进本校派出督导及时更新最终联系代码。

（6）配合中山大学社会科学调查中心的日常数据核查，及时将问题反馈给访问员，并督促访问员修正访问行为；配合中山大学社会科学调查中心完成村居结束时的核查，确保数据核查没问题之后，调查队伍才离开调查村居。

（7）与调查员共同讨论工作分配和时间安排，确保调查工作进度及质量。

（8）帮助调查员解决遇到的各种问题，如寻找被调查户、解释问卷中的概念、对付难以合作的被访者等。

（9）如有必要，针对某些调查中失败的例子，如找错了家庭、没有完成调查、问错问题等，对调查员集中进行再训练。

（10）带队老师或督导员部分问卷进行陪访，根据中心反馈对问题访问员要加多陪访的次数，对其访问行为提供指导；对调查清单列表上已经完成调查的家庭进行入户或电话复核，每个每个村/居委会复核 5 户家庭，并填写好复核登记表。每个村/居委会陪访家户率为 8%，3 户；每个村/居委会复核家户率为 15%，5 户。

（11）完成村/居委会问卷，并完成针对村委会历史的半结构访谈，整理社区资源图，座谈录音及座谈会报告。

（12）负责调查礼金的管理与发放，调查回执与接触登记表的回收。

第五章　访问技巧

这一章会教给大家很多关于如何在与受访者接触中取得良好的效果、如何同受访者进行交谈以及获得他们许可和配合的一些细节、策略和技巧等，主要包括如下三方面内容：其一，如何入户：将教给大家如何确认受访者在家以及如何进入住宅的技巧和策略。其二，取得联系：将教给大家怎么做才能接近受访者并与之交谈的最好方法，能够使受访者感到愉快，并且同意接受参加调查。其三，吸引受访者的注意：如何与那些一开始存在疑虑并且不愿意参加访问的受访者打交道的技巧。

5.1 顺利通过大门

1.和村/居委员会接洽

在和住户接触前，非常重要的一点是和当地村、居委员会取得联系。这样可以提升作为一名专业访员的合法性。此外，这也有利于当附近居民打电话询问有关情况时，当地村、居委员会就可告诉询问者访员的合法身份，而非推销员或来附近作案的人。

执行督导员应将事先把“致村、居委员会的一封信”传给当地居委会或村委会，并事先提交当地访问的访员名单，通知他们访员在当地的出现和目的，并委托村/居委会在公告栏贴出 CLDS 调查告示。公告示例如下：

关于近期“中国劳动力动态调查”的通知

从_____月______日开始，中山大学社会科学调查中心调查员将会在本村进行“中国劳动力动态调查”，为期 30 天左右，请各户配合！

XXXX 村委会

2018 年____月____日

图 1 村/居委会调查公告

当和当地居/村委员会接触时，访员可以打电话或者亲自去拜访样本家庭户所在辖区的居委会或村委会，确信记下干部的姓名。在拜访村/居委员会时，要表现的像一个专业人士。同时，随身携带身份证和中心访员证，以提升访员的合法性。

2.入户调查的时间

打电话联系住户的最好时机是什么？研究表明晚间是和住户取得联系的最有效的时间，特别是那些很少在家的人，周末是其次的时间段。

**星期天－星期四的晚上**	**晚上 6 点－晚上 9 点**
**星期五－星期六的晚上**	**晚上 6 点－晚上 9 点**
**星期六和星期天全天**	**上午 9 点－晚上 6 点**
**星期一－星期五的白天**	**上午 9 点－晚上 6 点**

总的来说，如果不确定能和住户取得联系的最佳时间，在实际操作中，晚上拜访是最经常使用的，其次是周末访问，最后是工作日联系。

在一个地区，和邻居交谈是非常有用的，可以询问和住户取得联系的最佳时间。必须牢记的是，对待所有的住户，访员都必须时刻保持自信。和邻居交谈的时候，可以告诉他们自己是来自于 XX 大学，但是必须小心不要泄露受访者的姓名以及联系受访者的原因是什么。

不要在短时间内“过分接触”任何家庭，这一点也很重要。如果访员在与某个人在其家中接触时，出现了一些临时的状况，留出足够多的时间，等待一些变化，并避免出现“骚扰”之嫌。

3.访问路线规划

要留下充裕的时间计划好工作，在地图上标出行动路线。最好从那些最难接触到的人居住的地区着手，但是也不应忽视从这些地区来回时路过的部分。还有，先从那些最容易受到天气状况影响的地区开始，要考虑到春洪、大雪等可能阻断偏远地区交通的灾害。

4.封闭式住宅楼的应对

有些受访者可能居住在有进入限制的地方，如有保安的公寓或公寓综合建筑、有门禁的社区等。遇到这种情况，都会为与居住于此的受访者接触，带来更大的难度。然而我们发现，通过良好的观察和计划，与适当人员的接触，很少会有无法克服的问题出现。这里有一些可以帮助访员顺利进入这些地方的技巧。

a.始终在显著位置佩戴调查证件。

b.多带些项目介绍的信函和研究说明书。

c.寻找提到楼房管理者或所有者姓名的标示牌。

d.告诉保安或门卫自己并非来是推销人员或拉客的，而且不会挨个骚扰住户。访员应该解释自己的身份，出示身份证明，并且告诉他们受访家庭已经在等待自己。

e.如果有必要与楼房管理者交谈，强调一下研究的重要性，以及保护隐私和机密的最严格要求。告诉管理者，访谈是完全自愿的，受访者有权自己决定是否参与研究。

正如前面提到的那样，调查现场每一种情况都具有一定的特殊性，每一种可

能的途径都应该去尝试一下。在不同的场合，使用的方法会有很大差异。在大多数情况下，访员应当收集所有可能收集到的相关事实，然后及时和督导打电话沟通，讨论一下行动计划。

5.如何突破僵局

在接触之前，访员应做好充分的准备。一般在初次接触时，常遇到僵局的情况，遇到这种情况，需要必要的接触技巧，以下是一些经验分享：

（1）狗

尤其在黑夜时，狗吠之声不断从区内传出来，这时的应付方法可如下：
到恶犬拦路，访员应留意犬是否抬头向天长“啸”，如是者，这不过是它提醒其它犬只，有陌生人进来而已。若犬只尾垂兼慢慢后退，则表示它已胆怯，访员可直视犬目光，大踏步而过。

若恶犬尾垂但裂嘴露齿发出“呼呼”声，则表示它随时扑过来，面对这种情况，走，并不是办法。访员应以炯炯的目光，直视着拦路恶犬，而恶犬被盯视，尾巴慢慢有点无力的往下垂而没有任何动作时，这表示恶犬已有点胆怯，即可向前踏一步并迫令它往后退。

（2）被门闸阻隔

若被访住户邻近有相熟的街坊，而他们又与闸内的街坊很相熟时,可请他们代为引领；也可以先访问邻近街坊,在完成访问后即说：“呀,你认识这闸里面的街坊啊，平时有没有同他们打交道呀，我想问他们几个问题呢，我又不知道怎样称呼他们，我看你可不可以帮我代为通传，说是中山大学访员，专程来探访的。”很多时，这位街坊会乐意代为引领。

（3）怀疑你的身份而不开闸

给你吃闭门羹又应如何？找相熟的街坊,代为引领,或在门外高声介绍自己、工作机构及访问的目的，以缩短下次访问时的社会性距离。当然，有女访员同行访问，会减低对方的怀疑。

（4）拒绝开门

在你敲门或按门铃时，受访者在观察孔看一下后不开门。这时你应该继续敲第二次门，事实上差不多有一半人在第二次敲门的情况下开门。如果他在你第二次敲门后仍不开门，我们要求你第三次敲门。经验显示，三次敲门使 90%以上的原本拒绝开门的人开门。如果连续三次敲门仍不开门，这表明住户家中无人，或

者此时住户因某种特殊原因（心情不好，有重要事情在做，家中只有老人或儿童在家，不便给陌生人开门等情况）而不便开门，这时我们可以按一次未遇处理，继续访问下一家住户，一定时间后进行回访。

（5）开门后便想关门

这种情形反映对方有怀疑心，但不是很强烈，可能是别的原因驱使他想关门，你不妨轻轻及很自然地向前踏上一小步，微微使对方的门关不上，随后脸上带着微笑望着对方，很有诚意的向他介绍自己、工作机构和调查目的，促使对方无暇怀疑及潜移默化接受你的访问。

（6）太忙了

开门听取你的解释之后，调查对象表示太忙或不感兴趣。在这种情况下，你要再次说明这次调查的重要性，而且只是要他说一些有关他的简单想法。有一种方法被证明十分有效，访问时对调查对象说："事实上我们要了解的是一些本来就有的想法，内容非常简单，比如你是否觉得现在物价上涨非常严重……"这样一下转入到问题上，许多调查对象往往一下子不自觉地被带入访问。

大家要坚持在态度并不友好的家庭中进行访问，是因为他们往往是代表了居民中的一个特殊的群体（通常是收入比较高，职位较高的人），如果我们轻易放弃了对他们的访问，则有可能使我们得到一个偏差很大的（缺少这一群人的意见）的调查结果，则样本总体的代表性会下降。

另外，一些访员在遇到拒访时会有受到挫折感而放弃对这种家庭的访问，有一些访员就觉得这样没面子或有些厚脸皮。我们需要请你清楚的是：对于一个访员来说，你最高的职业标准是成功地实施对特定受访者的访问，并为此付出你的耐心和智慧，你的能力不在于能访问几个对你友好的家庭，而在于你能够应付那些对你并不友好却最终接受你访问的家庭。

（7）开了门后不理不睬

这是不要紧，访员就以对方所专注的事情为话题，如对方正在做家务，就从谈家务开始，转引访问。

（8）开了门后就埋怨

访员应站在他的位置上，分享他的苦恼，留意他的表情、动作、感受及总结他的感觉，接纳他的理念及想法，从而提出一些可行的解决途径，并表示访员可在这方面，给予协助。

6.成功实现接触

（1）访谈的成功途径

一旦访员到达一个地区，面带微笑去拜访每一户家庭，并且相信居民是容易打交道的人。开门的人的反应可能混杂着好奇心与正式的礼貌。这种最初的兴趣，将给访员时间来表明身份，以排除推销员、催债人或其他户主不愿意与其交谈的陌生人的可能性。除了证明自己是中山大学社会科学调查研究中心和中山大学社会科学调查中心的访员的身份后，拿出随身携带的印有访员单位的蓝色包和一份签署过的隐私声明等资料，就会发现这是非常有效的。访员必须与开门的人建立起协作关系，以便获取所需信息。

（2）如果无人在家

如果发现没有人在家，留一个便条是一个不错的方法。有多种手段可供访员在留条的时候使用。最常用的便是“对不起我来的时候你不在家”卡片。便条的内容应该包括你拜访的日期和时间，一段简短的信息，以及中心的联系电话 020——84113169 和访员的联系电话。再次强调，注意保护受访者的隐私，即使邻居拾到了卡片也不会得知访问的详细情况。

如果邻居家有人，可以向他们打听一下。如果没有得到任何信息，在同一天的不同时间，同一个星期的不同日子或者周末再来。

（3）自我介绍——在第一次接触时

良好的初期接触，正确地介绍自己是工作成功的一半。自我介绍时，首先要在一两句话中表明身份、说明来意，语速不宜过快但要流畅，声音要清晰，音量要适中。初次见面，说话一定要温和客气，有礼貌。自我介绍可以同时递上访员证以表示真诚地访问，而非推销产品，也能解除受访者的戒心。对于受访者的质询应着重解释调查什么、为谁做此调查、保证其提供资料的保密性。入户后，寻找合适的位置坐下，坐的位置最好是受访者的左手，与调查对象成 45 度，这样既便于出示问卷和卡片，又便于记录。

当确实有人在家时，介绍自己和研究，是访谈的一个非常重要的环节。声音和措词以及面对面访谈时的衣着，都传达着访员可信度的信息。应当给人以认真严肃、令人愉快和自信的感觉。慢慢地把话说清楚，以避免产生混淆，并且给访谈定下基调。如果连自己都不想显得过于严肃，那么受访者显然更不愿意如此。在最初几分钟的接触中，必须确定受访者明确以下四点：

a.你是一个专业的访谈者；

b.你在为一家合法且有着良好声誉的机构而与他们接触；

c.你在收集重要且有价值的研究数据；

d.受访者的参与对研究的成功是至关重要的。

下面提供两个自我介绍的示范：

您好，我叫×××，我是××大学的学生。我们正在进行一项非常重要的研究，叫中国劳动力动态调查。最近您应该已经收到过关于这项研究的介绍信，让我向您简单地说明一下。

自我介绍不仅仅在最初的接触中很重要，在随后的接触中也同样重要。在对话中，始终积极地聆听，以便能及时就对方提出的问题和关心的东西进一步作介绍。

永远不要以可能被拒绝的方式提出要求。

例如，与其这样问：

“我现在能与你交谈吗？”

或者“现在是进行访谈的合适时间吗？”

或者“现在可以开始了吗？”

不如这样说：

“我希望现在对你来说是一个合适的时间。”

或者“有人告诉我现在这个时间对你来说是很合适的。”

要一直相信此时是做访谈的最佳时间，访谈可以在接触的时候进行。

进行访问时相信受访者是友好而且有兴趣的。如果受访者表现出不友好或漠不关心，那是因为他们还不知道为什么访员要来拜访。访员的心理状态常常会影响受访者的反应。如果访员的谈话是不确定或紧张的，这种感觉会传递给受访者，受访者就会受其影响。如果访员使用的是一种令人愉快、积极而又倍感亲切的方式，这也会影响到受访者的态度。清楚自己在做什么、怎么做，这会提升在受访者心目中的可信度。认真地聆听受访者说的内容、语气、潜台词，然后据此回应。与他们交谈，而非向他们唠叨。如果他们相信访员对他们感兴趣，他们才会更愿意参与进来。

准备好回答问题。不同的受访者会有不同的关切点和问题。访员必须时刻准备好以平静而专业的态度，给出正确而有礼貌的回答。要做到这一点，首先必须

尽量多学习与研究有关的东西，并且自己组织好解释。一些受访者可能只需要一些关于访谈目的的简单解释，另外一些则可能想知道更多的细节。准备好若干个不同的解释和步骤，这样就可以根据受访者的要求进行调整。一旦问题回答完毕，不要等待他们暗示才接着说，要直接进入访谈或者受访者的问题，而不必停顿或犹豫。如果他们还有更多的问题，或者需要了解更多细节，他们会向访员询问的。

5.2 进行预约

通常，受访者不会立刻完成访谈，所以需要与他预约之后再来。当遇到这种情况的时候，把下面的技巧记在心中：

一定要给现场访谈留出充裕的时间。往往提前计划好访谈是非常重要的，但受访者也会轻易的打破这一局面。

如果要访员进行一次预约，那么尽量在早晨工作开始的时候，以便能在这个访谈后再进行其他的访谈，这样访员就不会出现在一个访谈进行中的时候突然发现自己本应该在另一个受访者那里的情形了。

如果两次预约之间的时间不够进行另外一次访谈，要准备好在这段时间里做其他工作。

不要使用电话作为调查的最初接触方式，这使得受访者极容易拒绝访谈。不过，如果受访者建议这样做，就可以使用电话作来确认预约。、

如果准时到了受访地，但是没有人在家，留一个“对不起我来的时候你不在家”卡片。如果能感觉受访者虽然有意合作但却心不在焉，应该留下电话号码让其打电话改约一个合适的时间，附上一些诸如“我会在星期四和星期五再来你家附近”这样的话。不过，在“对不起我来的时候你不在家”卡片上留下自己的家庭电话时，要谨慎一些。

5.3 建立融洽关系的技巧

要在访谈中取得成功，每个访员必须培养起自己独特的风格，然后与潜在的受访者以某种使人愉悦的方式互动。个人风格应着重体现一种专业感觉，并且让自己与受访者建立起一种和谐融洽的关系。下面是一些关于培养自己访谈风格的

技巧。

a. **专业性使访员与众不同：**社会科学调查是收集数据的一项高深专业技能。

b. **努力保持一种令人愉快的友好态度：**时刻保持积极态度。切勿与受访者争论，这是很重要的。

c. **吸引对方的注意力：**现在人与诸如民意调查、市场调研、商场调查和电话调查等打交道的次数，超出了他们的愿意程度。他们只在预期会获得利益时才会听访员的建议。所以访员应该提供一些关于此研究重要性的信息，并且解释清楚研究的结果会如何使用。

d. **建立对话：我们都喜欢愉快的交谈。**你说，我听；我说，你听；我们分享信息。受访者需要相信他们的观点是有价值且正确，访员理解了他们所要表达的全部重点。要想取得这种效果，访员必须提供必要的信息，复述受访者的意见，并将这些意见或重点结合起来进行更深入的解释。对话不应太长，才能更有效率。

e. **建立起对受访者的认知：**每次访员与受访者接触时，都会更进一步了解他们。了解他们的日程表，以便知道拜访的合适时间。了解他们的家庭或工作事务。在了解一个特定的受访者后，访员就会知道如何向其解释调查对其生活的重要性了。

f. **想好一些常见问题的答案：**受访者总是有许多方式来问"为什么是我呢？"这类问题。访员的任务不仅仅是预先想好一些常见问题，还需要知道如何提供给他们易于理解的答案。认真聆听受访者的用语，如习语、语气、语速等。这可以提供一些线索，来帮助访员以一种更容易引起共鸣的方式回应受访者。

g. **使用积极的聆听手法：**聆听是一门艺术。在人们说话的时候，他们并不需要意见、建议或评价，他们只需要有人聆听。所以，认真地聆听吧，不清楚的地方要提出来，然后复述一下。使用受访者的语言来解释。下面是一些特定的技巧：

不仅要聆听他们的问题，也要聆听他们的意见。

注意受访者的语气和停顿。

在回应受访者时，应当带着对其讲话的尊重。

找出受访者停顿犹豫背后的原因。

使用受访者的措词。例如："我理解你不感兴趣，但让我来解释……"

h. 每次接触都是独特的：十个人看同样的事情，会有十种不同的看法。每次与受访者的接触，都是他们生活的一个片段。把每次接触都当成七巧板的一块。

每次接触都创造了七巧板中新的一块，受访者的情况就变得很清晰。

适时是关键：受访者很想知道他们在被要求做什么、什么时候做、需要做多久。虽然有时候，要求看起来像是对他们生活的滋扰，但访员的任务就是创造一种便利的方式来使受访者同意安排时间接受访谈。

i. 在积极的气氛中结束：只要受访者知道访员将要离开的时间，一般都会同意。如果访员感觉出了厌烦和抵抗的情绪，那么轻松地离开，等待改天再约。

j. 为再次接触铺好垫：如果确实没法说服受访者在某次拜访中接受访谈，访员可以说："我会在你不那么忙的时候再联系你。"或者"下次我到这附近的时候再来。"这些话给再次接触铺好了垫，因为它们暗示了访员对他人现在不能接受访谈的理由的尊重。

保密：再次强调这项研究的保密是有必要的。举例而言，答案只以整体的方式出现，个人姓名永远不会出现在答案上面。

时刻强调每个受访者的重要性：应该让每个受访者都觉得自己对于整个研究的成功所起的作用是至关重要的。受访者应该觉得自己是独特的，也就是说，由于整个样本系统的精确性，每一个被选择的受访者都是无可替代的。

第六章　数据质量概述

一般来说，数据质量即统计数据对用户需求的满足程度，主要包括准确性和有效性，有时也会包括适用性、及时性、可比性和可获得性等等。其实，整个社会研究的根本目的就是为了减少误差，以获得准确、可靠而又有效的统计数据，而对数据质量的要求必然牵涉到社会研究的整个过程，因为包括研究设计、问卷编制、抽样、调查实施、数据收集和数据处理在内的所有过程，都可能影响到数据的质量。所以说，对于数据质量的控制不仅是方法论的问题，而且也是调查实践层面的问题，具体就是管理层面的问题。此处略去影响数据质量的研究设计、问卷编制、抽样、数据处理等方面，只取其狭隘的一面，即在入户问卷调查的实施过程中，如何保障数据的质量。问卷调查的实施过程中，主要有三个方面会影响数据的质量，即调查员、被访者和调查的环境及时间。

6.1 调查员的因素

6.1.1 调查员影响调查质量的分类及原因

由调查员的差错造成的误差主要有四类：（1）询问误差，即调查员在询问时对问卷题目有所更改或省略等造成的被访者的回答误差；（2）追问误差，即调查员经由不适当、不必要、不相干甚至有偏见的追问方式造成的误差；（3）记录误差，是调查员在听、理解和记录被访者的回答时造成的误差；（4）欺骗误差，由调查员伪造部分或全部答案而造成。

按照 Floyd（1990）的理论，问卷调查应该是在一个拟定的标准化下，找出或估计出社会现象及其发生程度。所谓标准化，就是使每一被访者面对相同的问题、相同的提问及相同的答案纪录方式。只有这样，收集的资料才有可比性，具有关于这类现象的社会学统计意义。换句话说，收集资料所显现的差异才可以被正确解释为受访者之间的差异，而不是调查过程的差异。

标准化调查的关键是，当被访者对调查内容存有疑义时，调查员们应该有基本一致的处理方式或答复口径，如果调查员发现被访者对题器内容不明白或有疑惑，而自作主张诠释题意，或修改题目陈述，则可能导致这样的结果：一些被访者被问了这个意思，另外一些的被访者则可能被问另一层意思。这样收集到的资料，就是一个缺乏信度的资料。所以标准化的、无诱导的调查过程是必须的，调查过程中所有对调查员的控制，都紧紧围绕“标准化、无诱导的调查”而展开。实际调查中，调查员自作主张改变题意的现象时有发生，究其原因主要在：（1）题目念起来很拗口；（2）调查员试图构建一种谈话式的、非正式的互动情境。出于这一目的，调查员在调查实施时往往极容易按照自己的理解，在互动的情境中对问卷题做这样或那样的主观解释，有意无意地加入个人的提示。有经验的调查员比无经验的调查员，更倾向于在调查中采取非正式的互动方式。

所以，除了调查员是否按规定程序和标准、是否有意或无意地对被调查者施加影响、记录的认真程度等，还有调查员的普通话水平和方言能力、调查员的心理素质都很不可忽视，此外调查员的其他特征也很重要，例如相貌、身高、衣着等应尽量与普通民众的平均期望值相接近，否则，会因为这些因素引起被访者的心理波动，从而导致不期的调查效果，影响调查质量。

6.1.2 针对调查员可能影响调查质量的策略

1.培训控制

调查前的培训应该是充分又适度的。“充分”即在于，培训中应尽量让调查员熟悉问卷资料和研究者的调查意图，尽量估计到调查过程可能出现的方方面面的问题。所谓“适度”培训，即不过分要求。这与前述结论有关，因为过分熟悉调查过程，往往导致调查员过分相信或运用自己的调查技巧，可能出现客观偏离问题。

2.调查过程的控制

本次调查将采用计算机辅助调查，该技术有助于对问卷的漏答、错答、跳跃出错等进行严格控制，此外，数据将更为及时地反馈回到总部数据管理部门的人员手中，数据管理部门的工作是及时审核和发现问卷中出现的各种问题，及时发现，及时通过电话或者重新入户等方法给予纠正，既提高了工作绩效，又保证了调查质量，还对以后调查工作具有及时的警示作用，减少调查员作弊的可能性。此外，为了监控调查员工作的认真情况，总部的数据管理人员还将进行一定比例的复核，复核往往采用电话复核。即通过电话找到被访者，针对容易出现调查员自己填答的问题进行回访，以检验调查员是否作弊。复核需要注意的其他现象有：同一位调查员连续调查同类群体、连续出现拒绝留电话的被访者、连续出现同一类问题有不清楚、拒绝回答的情况等等，这类现象的出现往往预示调查员可能作弊，或者表示调查员在问卷理解上有系统偏差，需要立即与调查员沟通。最好的沟通方式是与调查员及时面谈，先听他对问题作解释或介绍调查情况，然后落实是否与实际情况相符合。复核中对调查员质量控制的另外一个重要办法是实地复核，即由当地督导或专门的复核员到调查现场，对既定的问卷填答逐一进行复核。一般而言，复核员应该与调查员之间没有任何联系，属于背对背进行的复核。

3.调查员的自主权

理论上，不同调查员在自主控制范围内的不同表现，常常是他们收集资料出现差异的主导原因。那么，怎样的言谈举止才称之为合适呢？回答自然是以保证数据质量为参照，但这一点又完全人云亦云，没有确切的测量标准。根据以往经验，可以让渡的空间包括：调查开始时间、调查开始前的互动、调查时的选位、调查之后的互动。调查开始的时间，多半由调查员自行与被访者或与被访者的中

介（如居委会干部等）直接取得联络，并在联络中相互约定访问时间。这种事前的约定，相比调查员敲门入户实时开始的访问，更有助于访问双方建立最初良好的信任关系。

调查员按约定入户，在正式调查开始前，应该与被访者之间有一段进一步沟通的时间，以活跃访问气氛。即进入被访者家中，利用二三分钟时间彼此介绍、寒暄或谈论某些兴趣话题等都非常必要。至于如何沟通，谈哪些内容，应随被访者的家庭环境、职业身份的不同而不同，同时也依调查员的个人性情、兴趣及爱好而相异，并不存在严格一致的规定和要求。

调查中互动双方的选位亦相当重要。选位的标准是使双方便于交流，同时应首先考虑尊重对方。但选位时是否应尽量避免被访者看到问卷内容，学者们对此存有不同看法。一些学者认为，入户调查所采取的面对面互动，应该采取调查员读题提问、被访者回答的方式。整个过程都是调查员主动、被访者被动答题的过程。被访者无需阅读问卷，因为他们的阅读，很可能使其回答不具真实性而随问卷的内容场景作某种修正式的回答。但另一些学者则认为，只要问卷设计合理、规范，为取得被访对象的信任和他的进一步理解，也为杜绝调查员读题时的主观引导，完全可以敞开问卷给被访者作出他的判断或选择。究竟何者、何种位置选择较为合理，同样因人因境而宜。

调查完毕，必要的谢辞除了能够显示人际互动的礼貌之外，还可能在陌生人沟通的最后时刻给被访者留以舒适的自省空间。

4. 研究者与调查员的互动

由于调查员直接同被访者进行互动，许多无法反映在问卷上的，但又可能与研究内容相关的信息，可以通过与调查员的互动来获释；同时，重视研究者与调查员的互动，有助于帮助调查员建立良好的专业知识和心态。

调查过程经常会遭遇各种意料之中或意料之外的情境，这些情境对调查员心态的影响不尽相同，有正面也有负面。这就需要负责人通过与调查员的互动去调整他们的心态。此外，研究者与调查员还可以就以下几点进行交流：问卷设计内容是否合理、调查质量或调查效果如何、研究目的是否能通过调查资料反映等等。通常情况下，研究者对某一调查员反映的情况，需要作代表性考证。即单一一位调查员接触的被访个案有限，他反映的情况未必具有代表性。但假如连续多位调查员反映同一问题，则该问题可能具有代表性了，应引起研究者的足够重视。

6.2 被访者的因素

问卷调查过程是被访者一连串认知活动的表现，被访者基于对调查员所述问题的了解，依据自己的知识，作出思考和判断，最后给出答案。所以，从调查结果上考察，调查过程中的被访者行为一般有如下四种类型：（1）拒绝参与调查；（2）同意参与调查，但是拒绝回答问卷中的某些问题；（3）同意参与调查，并且回答了一些问题，但出于私人目的或者利益的驱动，回答过程中刻意隐瞒某些事实，使得答案系统地偏离了真实情况；（4）同意参与调查，充分配合，回答了所有问题。

第四种是调查中我们期望的理想被访者的行为。但不容忽视的是，大量出现的其他三类被访者行为将会给调查带来很大的误差。所谓调查中的被访者因素，可以定义为调查过程中被访者在问卷回答上偏离“真实答案”的任何可能的影响因素。这些因素包括问卷设计不佳、题目问法不对、被访者理解题意的差异、性格差异、教育程度高低、记忆谬误以及被访者故意回避问卷中有关敏感问题等等。在调查过程中，这些因素相互交织、相互影响的结果，具体表现为产生调查误差的各种被访者行为。

被访者回答的质量取决于被访者对问题的理解，有关信息的获得以及对以前经历或知识的记忆能力。由此可见，被访者自身具有的某些特质会影响他们对题目的理解及其回答的正确性或真实性。这里所说的真实性或正确性，并不必然暗示被访者故意为之，因为过往的调查经验亦已说明，调查过程中被访者对某些题目的回答所出现的偏差，可能是其对题目本身没有正确理解或对过去事件的不准确回忆造成的，但不可否认，某些不正确或不真实确有可能因为被访者出于某种考量而故意为之的。

6.2.1 性别与年龄

女性富有同情心，比较容易被调查员打动，她们一般比男性更乐意接受访问，并且被访过程中更加有耐心，配合程度也高。男性被访者拒绝访问的比例明显高于女性。可是，晚间调查，对家庭主妇的访问经常容易被打断，晚饭前她们忙于准备晚饭，晚饭后大多要收拾餐具和照顾孩子等，或闲坐下来，追看电视剧。所

以，即使她们愿意接受访问，也很容易被其他事情打断，或想其他琐事而无法集中精力于答题。

被访者的年龄对于调查质量的影响也是显而易见的。处在不同年龄段的被访者在调查过程中的表现是多种多样的，年长的被访者在调查中的表现尤为特殊。对年长者的访问，除了因其年龄大、耳朵背、反应慢等因素导致访问时间过长之外，问卷中的很多客观题目，诸如被访者父母的出生年份、教育状况、户口迁移、职业类别等，因为涉及填答的事件太久远，一些被访者早已对此无法记忆，因此出现了调查题目的空白。即使只是问被访者自己的职业经历，对这些长者来说也勉为其难，因为需要回忆的地方太多，很多记忆已经渐次模糊，甚至无法在记忆中唤醒。此外，尽管研究者在设计题目时已经避免了恶意或偏见，但是题中出现的一些回顾既往历史的叙说，对极个别被访者来说则可能勾起一段痛苦的回忆，引起被访者心理上的不舒坦或出现悲情万分的尴尬局面。在调查过程中虽没有引起被访者反感，没有出现因此拒绝访问的个案，但被访者却可能表示出不愿直面该问题的言语，或直接跳答，从而影响后续调查情绪和调查质量。不过，年长的被访者通常因为不需要工作，空闲时间比较多，对于调查员的来访非常欢迎，极容易将调查员作为闲聊对象而认真作答。

6.2.2 性格特征

一般来说，调查问卷既包括被访者基本情况等客观信息，也包括一些涉及对某些事件、说法、行为等看法或态度一类的主观问题。对于前一类问题，研究者通常会在设计问卷的时候加以考量，巧妙地在问卷的不同地方设置逻辑检验题，以甄别答案的真假。对于后一类问题，到目前为止除了强调问卷调查的普遍性指导原则之外，并没有行之有效的质量控制手段。对于此类主观问题，被访者提供正确答案的意愿往往因为性格差异、生活阅历以及利益驱动等因素而大相径庭。有的被访者出于社会期许的目的，在回答问题时，总是试图有意隐瞒真实态度，从而使答案偏离真实情况，造成“作答误差”。

一般来说，在多数的调查员与被访者的互动过程中，调查员应该是临时建立起来的陌生人互动场域的主题支配者，被访者则处于被支配地位，调查员的主导作用只有在被访者的积极配合下才得以实现。但现实中，被访者的配合有程度上

的差异。除一种“刀枪不入”的拒访者、一种“百分之百”的服从者之外，绝大多数的被访者都是有限度的配合者。这种限度往往视被访者的性格、地位及所处环境的不同而不同。自主型被访者容易在与调查员的互动过程中“反客为主”，占据支配地位。与自主型或主动型被访者相比，依赖型、顺从型或被动型调查者更容易造成调查受阻。这类被访者有的表现为对答案的不自信，尤其回答态度题目时总是犹豫不决、模棱两可，感觉调查员说的这也对，那也对，没有能力给出最符合自己想法的答案；有的表现为缺乏思考，或存在思维惰性，对以表格形式出现的态度行为题往往倾向于某个固定的选项；有的把访谈理解为是家庭行为，而由于其在家庭中的角色不是主导型的，或者说在家庭事务中不发表意见，在回答问题时常常要征求在家庭生活中扮演强势角色的家庭成员的意见。

调查经验还发现，在进行开放题的访问时，如果遇到健谈的被访者，访问通常比较容易进行，并且可以收集到更多的资料。而在封闭题中，寡言的被访者则相对容易调查，健谈的被访者常常扯开话题，导致访问时间延长。但从总体来说，健谈的被访者更容易接受访问，访问过程中的配合程度也较寡言的被访者高。

6.2.3 主观障碍

社会调查中有些特定的话题一般很难得到准确答案，它主要包括“令人难堪的”问题、“涉及敏感”的问题、“有威胁性”的问题和“有挑衅可能”的问题等几个方面。这些问题之所以难以获得被访者的配合主要是因为侵犯隐私，有向第三者披露答案的风险以及出于答案的社会期许等几个因素的考虑而使被访者感到担心或者焦虑。

随着经济水平的提高，生活的多元化，社会环境的日益复杂以及生活节奏的加快，被访者在接受调查时的隐私观念和戒备心理也日益增强。那些对于被访者非常隐私或者是敏感的问题，通常会对被访者造成一种“主观障碍”，被访者担心如实提供信息会给自己带来麻烦，因此在心理上会对这种问题产生抵抗情绪，结果是不回答这个问题或者是考虑很久以后才给出答案。当然，随着调查员与被访者之间的互相信任建立之后，这些顾虑会有所减少。

社会期许也是被访者最后“生成”答案的一个重要考虑因素，它包括两种情况。第一，被访者希望获得调查员的认同，尤其是当一位男性被访者面对一位女

性调查员的时候，当他表达观点或暴露他的问题时，会不自觉地考虑到“面子”问题。尤其是当所问的问题会暴露被访者软弱的一面或是社会不兼容的行为时，他们感觉失面子。为了避免失面子，他们就以粉饰经历的陈述来重新解释自我行为，或者拒绝回答此类问题。第二，性格特征也可能导致被访者扭曲他们的答案，因为他们希望自己的想法或行为获得社会公众的接纳。依自我表现理论所言，人们多将自己最光辉的一面介绍给他人。在访谈过程中，被访者权衡再三的结果以及问题表述时的策略都将引起资料表达的偏差。这时就需要调查员在问到此类问题时再三强调回答并无对错之分，以及使用追问的技巧。

6.2.4 被访者生活经历

调查实践揭示，有过被访经历的人再次接受访问的成功率会受前次被访情景的影响。首先，前次调查中的调查员态度是决定此次是否接受调查的关键因素。如果上次调查经历愉快，调查员友善、诚恳，给被访者留下良好印象，那么再次接受调查的可能性大大增加。其次，前次访问内容的理解难易程度，访问时间长短，会影响被访者是否再次接受调查。一般来说，如果被访者感觉调查内容太难，超过自己的知识范围，或是自己不感兴趣的题目，或占用自己的私人时间太长，都会增加他对调查的厌恶情绪，从而拒绝受访。第三，前次访问内容与自身关系的密切程度也会影响被访者再次接受访问的可能性。调查实践表明，对于同样的问卷，曾经参与调查的被访者，比从未参与调查的被访者，更容易接受本次的访问，访问成功率也高。这可能因为前次受访经验的正向的影响。这种经验在政府调查中，强化的作用更明显，因为在政府调查中，被访者会觉得自己的意见被关注。此外，一些曾经接受过访问的被访者，假如上次调查有礼品赠送，他会不自觉地比较礼品利益，甚或可能不接受无礼品、少礼品的调查。此外，被访者的阅历对于他们回答问题的质量影响也很大。当问卷内容远离其生活经历时，被访者对于调查可能会有无所谓的态度，甚至漠不关心。

总之，对被访者的问卷调查质量的影响来自主客观两个方面的原因。从主观看，被访者的合作程度、心理上的作弊心态都直接决定调查能否顺利进行；从客观看，被访者的回答能力对于通过问卷调查方法收集的资料所具有的信度和效度都有致命的威胁。此外，由于调查过程本身是一个调查员与被访者人际沟通的过

程，社会期许程度以及理解题意的困难程度会因被访者自身特征的不同而有所差异。

6.3 调查的环境及时间

调查是在一定的情景下完成的。不同的场景下，人们往往有不同的反映。所以探讨调查场景对调查过程、调查质量的影响是有实践意义的。

6.3.1 入户时间

为了接触到更多的受访家庭，很多调查员都需在晚间入户进行调查，某些被访者看来这无疑是介入了他的生活，很可能打乱了他惯常的生活习惯，扰乱了他正在做的事情，或者是需要做一项花费他相当多时间和精力的事情，因此容易有排斥心理，进而影响调查质量。

不过，在调查实践中，调查员不得不在周一到周五的傍晚进行访问，原因在于大多数有工作的被访者白天都不在家，只有傍晚才下班回来。通常，他们在七点半左右吃完晚饭，然后看电视、或者与家里人在一起聊天。而五点半到七点半往往是做饭或者吃饭的时间，接受访问的难度增加，拒访率较高。但是如果此时被访者无须操持家务，那么接受调查的可能性也蛮高。晚上九点半以后，出于治安等诸多方面的考虑，调查员一般都会结束调查。所以从调查意愿上来说，刚刚吃完饭的被访者的合作意愿最高，其次就是准备晚饭的期间，最差的时段就是被访者一家正在进餐的时候，这时的合作意愿是最低的。

在周末，调查员则可以全天展开调查活动，因为理论上被访者一般是在家的。基本上，调查时间分为三个时段：上午九点到十二点，下午一点到六点和晚上七点到十点。但是在不同的地区时间存在差异。例如大城市则早晨起床的时间比较晚，而靠近农村的地区在上午八点钟入户已经比较迟了。北方入户的时间比较早，南方则比较晚。相应的，结束调查的时间，大城市、南方可以迟一些，但是小城市、靠近农村地区、北方则需要早些。

6.3.2 在场他人的影响

调查员反馈回来的信息是，在调查过程中，被访者的家人往往会参与调查。通常的情况是，如果被访者是男主人，家人一般不轻易插嘴；如果是女主人，男主人则往往陪坐一侧，女主人遇见某些问题涉及整个家庭的情况时，往往询问男主人之后才作答；如果被访者是家中的长辈，则几乎全家陪坐，代答的现象非常普遍；如果是晚辈，家长不会陪坐，但是时不时会前来察看，一旦问题涉及到父母或家庭的整体情况时，年轻的被访者通常需要咨询父母。所以，基本上可以看到这样一个规律性的作答模式：有关家庭的情况，被访者倾向于由家庭中拥有权威的人来作答；对于家庭其他成员的情况，被访者倾向于由该人亲自作答。

家庭其他成员参与调查的主要原因是：中国是一个家庭本位的社会，被访者个体是融于家庭的。因此，对家庭某一成员做调查时，不仅被访者本人的独立意识淡薄，个体本位的边界模糊不清，而且其他家庭成员也会义不容辞地将调查看成是自己的事情而积极参与。尤其涉及家庭利益的问题时，经常出现家庭他人与被访者共同作答的现象。某些问题无法排除家庭成员影响，这时我们建议应在问卷中加入环境变量。

问卷调查的原则之一就是避免他人干扰，因为这会严重影响到回答问题的真实性。但基于中国的文化传统以及调查过程中对组织资源的借用，这一点在现实中往往很难做到。为了调查能够进行，调查员有时不得不妥协。所以在重要他人在场的情况下，调查员应当具备适当的进攻性，一方面要能保证调查可以开展，另一方面又要尽量确保调查得到的资料不失真实性。为了避免村/居委会工作人员对被访者家庭受访过程的干扰，在有村/居委工作人员带领入户的情况下，应该在他们带领入户后让其离开，不用全程陪同访问过程。

6.3.3 每户问卷数量

每户家庭的调查都有家庭层面以及个体层面的问卷需要填答，这就导致家庭成员数量过多时，需要填答多份问卷。多份问卷的任务不仅给调查员带来繁重的任务要求，也对被访者家庭产生影响，一旦双方都产生心理厌倦或心理疲劳，都会影响调查过程中的数据质量。对于调查员来说，同一问卷，多次重复阅读、讲解，往往造成调查员在向被访者读选项时，不再以原题为序，而根据自己的经验按被访者可能回答的概率的高低作阅读顺序。追问时，亦可能根据自己前次的经

验，有意无意地提示或诱导被访者。同时，多次重复出现的调查，机械性开始显现，容易产生厌倦、疲劳，会让调查员产生快速将任务完成而不注重质量的心理。而对于被访者家庭来说，问卷数量过多，时间过长，也极易于影响其休息与劳务，造成情绪波动，对调查产生反感。此外，由于家庭成员并不总是同时在家，所以有时需要入户多次，同样会给一些家庭带去打扰，随着打扰次数的增多，家庭成员对调查的兴趣可能会有所下降甚至敷衍，进而影响需要被访问的个体。因此，在时间允许的情况下，应该尽量减少入户的次数，尽量在一两次内将一户家庭调查完。对于既要回答家庭问卷和个体问卷的家庭最知情的那位受访者来说，如果连续让其答两份问卷会使其感到厌倦和疲惫，因此在答完家庭问卷后可以在期间间隔一个被访者在让其继续回答个体问卷。

第七章　附录

附录 1：接触登记表

接触登记表是每一次接触样本户或者劳动力个体的接触情况记录。联系的目的包括做家庭问卷和做个体问卷两种。主要是记录下当时联系的情况，如果当时接触到了被访者，则要记录被访者的情况。由于有些家庭劳动力人口比较多并且时间不是很集中，可能需要多次联系，纸质版的接触情况一共可以记录 8 次。

中国劳动力动态调查（CLDS）“接触登记表”

F10 访问员姓名：___________________　　（编号：________________）
F11 问卷编号：_____________________
F12 户主姓名：___________________//99 无户主姓名
F13 住宅地址：____
F14 入户过程中是否有村/居行政人员引荐介绍？　1.有　　2.没有
F15 入户前是否发放预约信？　1.有　　2.没有

F16 第一次联系时间：____________月____________日____________时____________分
F17.1 联系的目的是：　1.做家庭问卷　　2.做个人问卷
F17.2 联系代码是_________
F17.3 接触人性别是：1.男　　2.女　　99.没有接触到被访户成员
F17.4 接触人的年龄是：
1.20 岁以下　　2.20—29 岁　　3.30—39 岁　　4.40—49 岁
5.50—59 岁　　6.60 岁以上　　7.无法判断
F17.5 接触人是否整洁：1.是　　2.否

F18 第二次联系时间：____________月____________日____________时____________分
F19.1 联系的目的是：　1.做家庭问卷　　2.做个人问卷
F19.2 联系代码是____________
F19.3 接触人性别是：1.男　　2.女　　99.没有接触到被访户成员
F19.4 接触人的年龄是：
1.20 岁以下　　2.20—29 岁　　3.30—39 岁　　4.40—49 岁
5.50—59 岁　　6.60 岁以上　　7.无法判断
F19.5 接触人是否整洁：1.是　　2.否

F20 第三次联系时间：____________月____________日____________时____________分
F21.1 联系的目的是：　1.做家庭问卷　　2.做个人问卷

F21.2 联系代码是____________
F21.3 接触人性别是：1.男　　2.女　　99.没有接触到被访户成员
F21.4 接触人的年龄是：
1.20 岁以下　　2.20—29 岁　　3.30—39 岁　　4.40—49 岁
5.50—59 岁　　6.60 岁以上　　7.无法判断
F21.5 接触人是否整洁：1.是　　2.否

F22 第四次联系时间：____________月____________日____________时____________分
F23.1 联系的目的是：　1.做家庭问卷　　2.做个人问卷
F23.2 联系代码是____________
F23.3 接触人性别是：1.男　　2.女　　99.没有接触到被访户成员
F23.4 接触人的年龄是：
1.20 岁以下　　2.20—29 岁　　3.30—39 岁　　4.40—49 岁
5.50—59 岁　　6.60 岁以上　　7.无法判断
F23.5 接触人是否整洁：1.是　　2.否

F24 第五次联系时间：____________月____________日____________时____________分
F25.1 联系的目的是：　1.做家庭问卷　　2.做个人问卷
F25.2 联系代码是____________
F25.3 接触人性别是：1.男　　2.女　　99.没有接触到被访户成员
F25.4 接触人的年龄是：
1.20 岁以下　　2.20—29 岁　　3.30—39 岁　　4.40—49 岁
5.50—59 岁　　6.60 岁以上　　7.无法判断
F25.5 接触人是否整洁：1.是　　2.否

F26 第六次联系时间：____________月____________日____________时____________分
F27.1 联系的目的是：　1.做家庭问卷　　2.做个人问卷
F27.2 联系代码是____________
F27.3 接触人性别是：1.男　　2.女　　99.没有接触到被访户成员
F27.4 接触人的年龄是：
1.20 岁以下　　2.20—29 岁　　3.30—39 岁　　4.40—49 岁
5.50—59 岁　　6.60 岁以上　　7.无法判断
F27.5 接触人是否整洁：1.是　　2.否

F27 第七次联系时间：____________月____________日____________时____________分
F28.1 联系的目的是：　1.做家庭问卷　　2.做个人问卷
F28.2 联系代码是____________
F28.3 接触人性别是：1.男　　2.女　　99.没有接触到被访户成员
F28.4 接触人的年龄是：
1.20 岁以下　　2.20—29 岁　　3.30—39 岁　　4.40—49 岁
5.50—59 岁　　6.60 岁以上　　7.无法判断
F28.5 接触人是否整洁：1.是　　2.否

F29 第七次联系时间：____________月____________日____________时____________分
F30.1 联系的目的是： 1.做家庭问卷 2.做个人问卷
F30.2 联系代码是____________
F30.3 接触人性别是：1.男 2.女 99.没有接触到被访户成员
F30.4 接触人的年龄是：
1.20 岁以下 2.20—29 岁 3.30—39 岁 4.40—49 岁
5.50—59 岁 6.60 岁以上 7.无法判断
F30.5 接触人是否整洁：1.是 2.否

附录 2：家庭联系代码

	访问员联系代码	（家庭）
100		开始问卷
110		上次因故中断，此次从上次中断处开始
120		中途拒访
210 没有联系上	2101	电话无人接听
	2102	电话号码错误
	2103	电话停机
	2104	敲门无人应答
	2105	无法进入建筑物/无法进入小区
	2106	受访户不住在此处，住在此社区
	2107	受访户不住在此处，不住在此社区
	2108	受访户不住在此处，不确定地址
220 联系到被访家庭，没有拒绝	2201	受访者本人愿意接受访问，但需要再次联系
	2202	受访者本人告知最佳联系时间
	2203	与受访者本人约定访问时间
	2204	其他家庭成员告知最佳联系时间
	2205	其他家庭成员约定访问时间
230 联系到被访家庭，拒访	2301	受访者本人拒绝访问
	2302	受访者本人强烈拒绝访问
	2303	其他家庭成员拒绝访问
	2304	其他家庭成员强烈拒绝访问
320 受访家庭不便调查	3201	受访家庭婚丧嫁娶不便接受调查
	3202	受访家庭有病人需要照顾，不便调查
330		特殊情况（请在备注中详细说明）
331		空户(在备注中详细说明,确认没人居住的住宅)
332		非住宅(请在备注中详细说明)
333		在建(请在备注中详细说明)
340		无法找到绘图地址(请在备注中详细说明)

附录 3：个人联系代码

	访问员联系代码	（个人）
100		开始或继续主问卷
110		上次因故中断，此次从上次中断处开始
120		中途拒访
210 没有联系上受访者	2101	电话无人接听
	2102	电话号码错误或是空号
	2103	电话停机
	2104	敲门无人应答
	2105	无法进入建筑物/无法进入小区
	2106	受访者外出，不在家
	2107	受访者旅游/出差
	2108	受访者住院
	2109	受访者住进养老院
220 接触了受访者，没有拒绝	2201	受访者本人表示愿意接受访问，但需要再次联系
	2202	受访者本人告知最佳联系时间
	2203	与受访者本人约定访问时间
	2204	其他家庭成员告知最佳联系时间
	2205	其他家庭成员约定访问时间
230 接触了受访者，拒访	2301	受访者本人拒绝访问
	2302	受访者本人强烈拒绝访问
	2303	其他家庭成员拒绝访问
	2304	其他家庭成员强烈拒绝访问
	2305	家人拒绝代答
320 受访者不能参加调查	3201	受访者照顾老人或病人而不便接受访问
	3202	婚丧嫁娶而不便接受访问
	3203	聋哑人
	3204	智障
	3205	精神病人
	3206	其他原因无法进行调查
	3207	严重口吃
330		特殊情况(请在备注中详细说明)

附录 4：督导确认代码

督导确认代码	（家庭）
501	完成调查
502	追踪家庭已搬出社区
510	受访者中途拒访
520	始终联系不上
530	彻底回绝
531	空户
532	非住宅
533	在建
540	无法找到绘图地址
550	特殊情况（在备注中说明）
督导确认代码	（个体）
501	受访者完成调查问卷（最终）
510	受访者中途拒访
520	始终联系不上
530	彻底回绝
540	被访者无法接受调查
550	特殊情况（在备注中说明）

附录 5：调查回执

回执是由被访者在访问后自行填答，回执的内容主要包括访问的时间、调查的主要内容、调查礼金的情况、被访者对访问的态度和被访者的家庭住址和电话等基本情况，一个家庭只有一份回执。回执的设计一方面是为了了解被访者对我们调查的基本态度，另一方面也是对访问员访问行为的一个有效的监督方式。

中山大学社会科学调查中心

家庭样本序号[___|___|___|___|___|___|___|___|___|___]___]___]___]___]___]___]

访问员姓名：________________

回　执

f.访问员是什么时间对您进行的访问？

_______年_____月_____日______点

g.访问员对您访问了大约多长时间？（请打√）

（1）2 小时以上（2）1 个半小时（3）1 小时左右（4）半小时左右（5）不到 20 分钟

h.访问员访问时，有其他人（除被访者之外）在场吗？（请打√）

（1）有（2）没有

i.有的话，是什么人？（请写出）_________

j.访问员有没有问过您家的住房情况呢？（请打√）

h.问过（2）没问过（3）记不清了

k.访问员有没有问过您家去年总收入的问题呢？（请打√）

（1）问过（2）没问过（3）记不清了

l.访问员有没有问过您家职业方面的问题呢？（请打√）

（1）问过（2）没问过（3）记不清了

m.您认为访问员在您家访问时的态度怎么样？（请打√）

e.非常认真（2）比较认真（3）不太认真（4）非常不认真

n.为感谢您的合作，我们委托访问员给您带去一份礼金。您收到礼金了吗？（请打√）

（1）收到了　　　（2）没收到

10、您对本次访问持什么态度？（请打√）

（1）非常欢迎　（2）虽然占用了时间，但还愿意接待　（3）太耽误时间，以后别再来了

11、您家一共收到_____(元）礼金。

其中家庭问卷礼金　______元，家庭问卷礼金签收人_______

其中个人问卷礼金**共**多少钱_______元；

个人问卷礼金签收人：______ ______ ______ _______ _______ ________ _______

12、最后，请写清您的住址、邮编与电话：

您家现在的住址：___________省_________市（县）__________区/ 街道/乡镇

_____________村委会 居委会

您现在住址的邮政编码：______________ 您或您家的电话：______________

附录 6：招聘流程

1、招聘目标与计划

招聘对象分为执行督导和访问员，执行督导负责各个调查地点的督导工作。招聘督导数量和访问员数量应依据调查地点的多少来定。因为 CLDS 调查中，每一个村居为 35 户，因此每个村居定 2 个访问员比较合适。执行督导一般是一个调查地点（一个市）设一个。为了能够选择更为优秀的督导员和调查员，一般面试比例最好设定为 3：1，即 3 个参加面试的人中间录取一个。督导员的要求则相对更高。

2、招聘方式

在需要招聘人数较多的情况下，可以通过人力资源网站、BBS、QQ 等网上渠道发布招聘信息；其他的发布方式通常还有海报、传单、学校或学院官方网站。在报名人数较多的情况下，为了减少面试的压力，可以预先进行一轮电话面试，主要是为了确定报名人在工作时间和工作地点上是否符合要求。

3、项目介绍（工作时间、工作内容，工作流程）

在招聘广告中，应该清楚地介绍“中国劳动力动态调查”项目的内容，包括工作开始的时间，工作的大概内容和工作流程。

4、招聘要求

负责任的态度，会调查地方言，能吃苦耐劳、较强的学习能力、会基本的电脑技能、office 基本技能。

5、面试

6、登录调查中心网站填写信息

各地全部确认的督导与访问员，都需要及时准备一份包含每一个参与人员邮

箱及手机号码的信息表，发回给中心，方便 CAPI 工作人员及时更新登录 ID 及密码。

附录 7：村居介绍信

关于协助中山大学开展
“2018 年中国劳动力动态调查”工作的函

为了解当前我国的劳动力状况，为社会建设提供决策依据，中山大学社会科学调查中心于 2018 年 7 月至 9 月期间，将开展“2018 年中国劳动力动态调查”。

此项调查的范围包括全国 29 个省（市、自治区）的 400 多个村（居）委会。根据全国统一的随机抽样，贵村被选定为调查点。中山大学社会科学调查中心聘请的工作人员等同志，将前往贵村（居）委会开展抽样与调查工作，请贵村（居）委会予以协助为盼！

感谢贵村（居）委会对我们工作的大力支持。

中山大学社会科学调查中心

2018 年 6 月 1 日

附录 8：家庭预约信

致“中国劳动力动态调查(CLDS)”受访家庭的一封信

尊敬的____________________:

您好！

为科学认识中国国国情，了解全国城乡居民的劳动力状况，为政府制定政策提供依据，中山大学社会科学调查中心计划在 2018 年 7 月至 9 月在全国开展劳动力调查。

本次在全国的 CLDS 调查一共将涉及 29 个省，400 左右的村/居，15000 多个家庭，30000 左右个人。经过科学的抽样，我们选中您的家庭进行访问。我们在访问过程中会有若干处随机录音，用于对我们的调查员进行监督，不做其他用途。为表达我们的谢意，访问完成后我们将赠送一份礼金。

我们的访问员将于_____月______日____点至____点期间，到您家里访问，请家中务必留人。感谢您的配合与支持！

我们的访问员都佩戴“中国劳动力动态调查访问员证”，您可以对他们的身份进行核实。调查中如有疑问，可以拨打下列电话：

本地调查督导电话：________________________

中大社会科学调查中心电话：020-84113169

再次感谢您的配合！

中山大学社会科学调查中心

2018 年 6 月 1 日

附录 9：督导复核问卷

中国劳动力动态调查（CLDS）复核问卷

家庭问卷编号[____|____|____|____|____|____|___|___|___|___|___|____|___|___|___|___]
受访者姓名：___________
访问员编号：____________
访问员姓名：______________
复核员姓名：______________
受访者电话：（家庭电话或手机）__________________

复核时间：_________年______月________日________时

您好，我是中山大学社会科学调查中心委派的复核员，前几天我们曾派访问员到您家做过有关劳动力方面的调查，为了保证问卷的质量，想跟您核实几个问题，耽误您一点时间可以吗？（复核员请出回答家庭问卷的被访者进行复核）

1、 接受复核情况：
1 □ 拒绝接受复核（**结束复核**）
2 □ 电话无人接听/无人开门（**结束复核**）
3 □ 电话空号（**结束复核**）
3 □ 接受复核

2、是否有访问员来您家做过劳动力方面的调查？
1 □ 有
2 □ 没有（**结束复核**）

3、请问您家一起住的有几口人__________；其中有血缘关系（婚姻）的家人有________位。

4、其中和您住在一起的 15-64 岁的人有________位（包括有血缘（婚姻）关系的家人和无血缘（婚姻）关系的其他人）？

4.1 其中和您住在一起的 65（包括 65 岁）岁以上的还在工作的人有______位（包括有血缘（婚姻）关系的家人和无血缘（婚姻）关系的其他人）？

5、访问员有没有询问过您家去年总收入的问题？
1 □ 询问过
2 □ 没问过
3 □ 记不清

6、访问员有没有询问过您家去年支出的问题？

1 □ 询问过
2 □ 没问过
3 □ 记不清

（复核员注意：如果被访者回答了个体问卷，则继续复核以下问题；否则结束复核）

7、访问员有没有拿皮尺为您量双臂伸展长度？

1 □ 有
2 □ 没有
3 □ 记不清

8、在访问您的时候，访问员是不是口读问题给您听的？

1 □ 是
2 □ 否
3 □ 记不清

9、访问结束后，访问员有没有给您礼金作为感谢呢？

1 □ 有，给了____________元。
2 □ 没有
3 □ 记不清

10、访问结束后，访问员有没有拿一份回执让您填写呢？

1 □ 有
2 □ 没有
3 □ 记不清

-----------------复核结束，感谢受访者-----------------

附录 10：督导复核报告表

访问所在省份___________ 督导员编号:__________ 督导员姓名:___________													
问卷编号	访问员姓名	复核日期月/日	复核不成功			复核摘要					是否通过复核	处理意见 1废卷 2补问 3重访 4其它——	补问或重访访问员的姓名
			无此住址或电话空号	家中无人或无人接电话	拒访	是否有访问员来过	家中人数是否有出入	是否有漏问问题	是否派发礼礼金	是否派发回执信			
		/											
		/											
		/											
		/											
		/											
……		/											

说明: 凡是填写是否选项的:1.是 2.否;

附录 11：工作流程

一、前期联系与准备

1、联系受访村/居委会
2、物资准备
A 纸质资料：绘图手册、调查手册、CAPI 手册、村居问卷、家庭问卷、个体问卷、入户接触登记表、拒访表、回执、村居介绍信、家庭预约信、访问员登记表、督导员登记表、调查小区抽样登记表（村/居）、调查小区抽样随机数表、绘图纸、住户清单列表封面、住户清单列表、随机数表 A 和 B 胸卡牌，U 盘
（由调查中心发电子版至各省，各地打印）
B、CAPI 资料：村居问卷、家庭问卷、个体问卷、接触登记表。
（由调查中心发至各省）
C、其他资料：绘图材料、皮尺、帽子。
（由调查中心统一配发 ）

↓

二、督导与访问员招聘

详见调查手册附录 6：招聘流程
注：各地尽量在村居联系得差不多的时候开始培训，以缩短培训完成后与开始调查的期间的间隔时间

↓

三、督导与访问员培训

一般培训 5 天，各地可以根据培训效果或培训人员掌握能力，适当增加培训天数。

↓

四、绘图

详见：绘图手册

↓

五、调查

详见调查手册：调查流程，访问员工作任务与督导员工作任务

附录 12：调查补充说明

一、督导复核

督导复核主要目的是对访问员的调查进行监督，发现整份数据作假，家庭人数作假，关键信息作假等问题。我们规定了执行督导每社区 5 份，外派督导每社区 3 份的复核量，主要是对调查进行监督。调查中心也会随机抽选部分复核过的家庭进行再次复核。

督导复核有两种方式，入户复核与电话复核。入户复核是在督导随访的过程中，随机选择该社区已经访问过的部分家庭上门进行复核，督导陪访的家庭也算。电话复核需要用到访问员回执，麻烦提醒访问员将纸质版回执上的电话填写完整，督导需要复核，首先要随机选择家庭，然后向访问员询问家庭电话，若纸质版的回执中忘了填写联系电话，可以让访问员打开家庭问卷中的联系模块查看家庭联系电话。对于追访样本，督导还可以通过系统查看样本中留的联系电话进行复核。

复核之前，督导要登入系统，查看这个家庭最终完成的个人问卷数量及姓名等信息；复核如果发现有问题，比如家庭人数，尤其是同住家庭成员人数，15-65 岁人数等出现问题，首先要根据系统中的信息向被访者确认是否理解有问题，如果确实存在问题，要向访问员确认具体情况。**如果确认访问员根本没有去那一户调查；或 15-64 岁同住家庭成员出现漏答，很有可能该样本全部需要重做，**所以提醒访问员一定作假或弄错。

复核出现上述严重问题的，麻烦立即将情况上报给中大派出督导，中大派出督导及时反映给调查中心联系人员，我们跟进修改及处理办法。

二、资料回收

除了最后的绘图资料扫描版，回执以及复核问卷也要回收，各执行督导需要检查信息是否填写完整，尤其是样本序号，家庭地址，签名等能够将纸质版的资料与数据对应的信息一定要填写准确。资料按社区整理后，邮寄地址为广州市海珠区新港西路 135 号中山大学东北区 344 栋，收件人为政光景　电话是 18319542958。
