# Supplementary material for: Impact of overwork on self-assessed health of rural-to-urban migrants: Limitations of work incentives moderation effect and industry heterogeneity
Source: PLoS One. 2025 Feb 14;20(2):e0317588. doi: 10.1371/journal.pone.0317588 (PMC11828385; doi:10.1371/journal.pone.0317588)
Supplement: S3 File — (DOCX) [file pone.0317588.s003.docx]

tab I1_14

drop if I1_14 >1 //筛选出当前是农业转移人口//

tab work2017 //2017年以来是否工作过//

drop if work2017>1

drop if I3a_8==1 //删除从事农林牧渔的人口//

tab birthyear

drop if birthyear>9999 //删除不合理的数值//

gen age=2018-birthyear

drop if age<0

drop if age>65

tab I3a_1 //工作小时数//

drop if I3a_1==99997

drop if I3a_1==99998

drop if I3a_1==99999 //删除缺失样本//

gen workhours=0

replace workhours=1 if (I3a_1 <=35 )

replace workhours=2 if (I3a_1 >= 36) & (I3a_1 <=55 )

replace workhours=3 if (I3a_1 >= 56) & (I3a_1 <=80 )

replace workhours=4 if (I3a_1 >= 81)

tab workhours

summarize workhours

sum workhours ,d

tab I7_5_1

drop if I7_5_1>999

gen totalhealth=1

replace totalhealth=0 if I7_5_1<4 //1为健康,0为身心俱疲//

tab totalhealth

summarize totalhealth //取标准差//

sum totalhealth ,d

gen totalhealthpf = r(skewness)

tab I7_3_11

drop if I7_3_11 >999 //对工作的整体满意度//

gen jobmanyi =0

replace jobmanyi=1 if I7_3_11<3 //1为满意，0为不满意//

tab jobmanyi

summarize jobmanyi //取标准差//

summarize jobmanyi,d

*******控制变量**********

tab maritalstatus

gen marriage=0

replace marriage=1 if maritalstatus>1 //未婚为0//

tab marriage

summarize marriage,d

tab Igender

gen gender=0

replace gender=1 if Igender<2 //男为1//

tab gender

summarize gender,d

tab I9_24

drop if I9_24>3

gen lasthealth=0

replace lasthealth=1 if I9_24>1

tab lasthealth //滞后一期健康状况//

summarize lasthealth,d

tab I2_1 //学历//

drop if I2_1>9999 //删除不合理的数值//

gen edu=1 //初中学历//

replace edu=2 if I2_1>3 //高中学历//

replace ed=3 if I2_1>7 //大学学历//

tab edu

summarize edu,d

tab edu

gen education=0

replace education=1 if edu>2 //接受过高等教育为1，否则为0//

tab birthyear

drop if birthyear>9999 //删除不合理的数值//

gen age=2018-birthyear

gen age2=age*age //年龄//

tab age

drop if age<0

drop if age>65 //删除年龄大于65岁//

summarize age,d

tab I3a_8

gen industry=0

replace industry=1 if I3a_8==2

replace industry=1 if I3a_8==4

replace industry=1 if I3a_8==6

replace industry=1 if I3a_8==7

replace industry=1 if I3a_8==9

replace industry=1 if I3a_8==10

replace industry=1 if I3a_8==13

replace industry=1 if I3a_8==14

replace industry=1 if I3a_8==15

tab industry

summarize industry

gen x1=jobmanyi*workhours

tab x1

bysort industry:tab x1

********************描述性统计************************

tab totalhealth

bysort industry :tab totalhealth

bysort industry :tab workhours totalhealth

tab workhours

bysort industry:tab workhours

tab jobmanyi

bysort industry:tab jobmanyi

tab lasthealth

bysort industry:tab lasthealth

tab gender

bysort industry:tab gender

sum age

bysort industry:sum age

tab marriage

bysort industry:tab marriage

tab edu

bysort industry:tab edu

tab lasthealth

bysort industry:tab lasthealth

pwcorr totalhealth workhours jobmanyi lasthealth marriage gender age edu industry ,sig //解释变量的相关性，检验内生性//

*************整体回归******************

logit totalhealth ib(#2).workhours,r

logit totalhealth ib(#2).workhours,or nolog

margins,dydx(*)

logit totalhealth ib(#2).workhours gender age edu marriage ,r

logit totalhealth ib(#2).workhours gender age edu marriage ,or nolog

margins,dydx(*)

logit totalhealth ib(#2).workhours gender age edu marriage lasthealth,r

logit totalhealth ib(#2).workhours gender age edu marriage lasthealth,or nolog

margins,dydx(*)

reg I7_5_1 ib(#2).workhours gender age edu marriage lasthealth,r

estat vif

statistics → binary-outcomes → postestimation → goodness-of-fit-after logistic/logit/probit //Hosmer-Lemeshow计算步骤//

*******************分行业异质性分析******************

bysort industry:logit totalhealth ib(#2).workhours gender age edu marriage lasthealth,r

bysort industry:logit totalhealth ib(#2).workhours gender age edu marriage lasthealth,or nolog

bdiff, group(industry) model(logit totalhealth ib(#2).workhours gender age edu marriage lasthealth) reps(1000) bsample

************加入工作满意度这个调节变量**************

******************将工作44小时作为界限***********

gen guolao=0

replace guolao=1 if I3a_1>44

gen x2=guolao*jobmanyi

tab x2

logit totalhealth guolao jobmanyi gender age edu marriage lasthealth ,or nolog

logit totalhealth guolao jobmanyi x2 gender age edu marriage lasthealth ,or nolog

margins,dydx(*)

bysort industry:logit totalhealth guolao jobmanyi x2 gender age edu marriage lasthealth ,or nolog

bdiff, group(industry) model(logit totalhealth guolao jobmanyi x2 gender age edu marriage lasthealth) reps(200) bsample

*****************将工作时间换为55界限****************

gen guolao1=0

replace guolao1=1 if I3a_1>55

gen x3=guolao1*jobmanyi

tab x3

logit totalhealth guolao1 jobmanyi gender age edu marriage lasthealth ,r

margins,dydx(*)

logit totalhealth guolao1 jobmanyi gender age edu marriage lasthealth ,or nolog

margins,dydx(*)

logit totalhealth guolao1 jobmanyi x3 gender age edu marriage lasthealth ,r

margins,dydx(*)

logit totalhealth guolao1 jobmanyi x3 gender age edu marriage lasthealth ,or nolog

margins,dydx(*)

reg totalhealth guolao1 jobmanyi x3 gender age edu marriage lasthealth,r

estat vif

bysort industry:logit totalhealth guolao1 jobmanyi x3 gender age edu marriage lasthealth ,r

bysort industry:logit totalhealth guolao1 jobmanyi x3 gender age edu marriage lasthealth ,or nolog

bdiff, group(industry) model(logit totalhealth guolao1 jobmanyi x3 gender age edu marriage lasthealth) reps(200) bsample

*********************当工作时间为80界限**************

gen guolao2=0

replace guolao2=1 if I3a_1>80

gen x4=guolao2*jobmanyi

tab x4

logit totalhealth guolao2 jobmanyi gender age edu marriage lasthealth ,r

logit totalhealth guolao2 jobmanyi gender age edu marriage lasthealth ,or nolog

margins,dydx(*)

logit totalhealth guolao2 jobmanyi x4 gender age edu marriage lasthealth ,r

logit totalhealth guolao2 jobmanyi x4 gender age edu marriage lasthealth ,or nolog

margins,dydx(*)

bysort industry:logit totalhealth guolao2 jobmanyi x4 gender age edu marriage lasthealth ,r

bysort industry:logit totalhealth guolao2 jobmanyi x4 gender age edu marriage lasthealth ,or nolog

*************************稳健性检验************************

reg I7_5_1 guolao1 jobmanyi x3 gender age edu marriage lasthealth,r

bysort industry: reg I7_5_1 guolao1 jobmanyi x3 gender age edu marriage lasthealth,r

reg I7_5_1 guolao2 jobmanyi x4 gender age edu marriage lasthealth,r

bysort industry: reg I7_5_1 guolao2 jobmanyi x4 gender age edu marriage lasthealth,r
